# Efficacy and safety of various primary treatment strategies for very early and early hepatocellular carcinoma: a network meta-analysis

**DOI:** 10.1186/s12935-021-02365-1

**Published:** 2021-12-19

**Authors:** Sha Yang, Huapeng Lin, Jianning Song

**Affiliations:** 1 Department of Surgery, Children’s Hospital of Chongqing Medical University, Chongqing, People’s Republic of China; 2 Department of Intensive Care Unit, Affiliated Hangzhou First People’s Hospital, Zhejiang University School of Medicine, Hangzhou, China; 3 Department of General Surgery, Guiqian International General Hospital, 1 Dongfeng Dadao, Wudang District, Guiyang, Guizhou 550018 People’s Republic of China; 4 Ministry of Education Key Laboratory of Child Development and Disorders, Chongqing, People’s Republic of China; 5 National Clinical Research Center for Child Health and Disorders, Chongqing, People’s Republic of China; 6 China International Science and Technology Cooperation Base of Child Development and Critical Disorders, Chongqing, People’s Republic of China; 7 Chongqing Key Laboratory of Pediatrics, Chongqing, People’s Republic of China; 8 Chongqing Engineering Research Center of Stem Cell Therapy, Chongqing, People’s Republic of China; 9 Children S Hospital of Chongqing Medical University, Chongqing, People’s Republic of China

**Keywords:** Network meta-analysis, Efficacy, Safety, Treatments, Very early and early-stage, Hepatocellular carcinoma

## Abstract

**Background:**

Several treatments are available for treatment of early and very early-stage Hepatocellular Carcinoma, also known as small Hepatocellular Carcinoma (SHCC). However, there is no consensus with regards to the efficacies of these methods. We aimed at identifying the most effective initial treatment strategy for SHCC through Bayesian network meta-analyses.

**Methods:**

Studies published between January, 2010, and February, 2021 were searched in EMBASE, Cochrane Library, PubMed and Web of science databases, and conference proceedings for trials. The included studies reported the survival outcomes of very early and early Hepatocellular Carcinoma patients subjected to radiofrequency ablation (RFA), microwave ablation (MWA), surgical resection (SR), transarterial chemoembolization (TACE), percutaneous ethanol injection (PEI), minimally invasive liver surgery (MIS), stereotactic body radiotherapy (SBRT) and cryoablation (CA). Then, data were extracted from studies that met the inclusion criteria. Patient survival data were retrieved from the published Kaplan–Meier curves and pooled. A Bayesian random-effects model was used to combine direct and indirect evidence.

**Results:**

A total of 2058 articles were retrieved and screened, from which 45 studies assessing the efficacies of 8 different treatments in 11,364 patients were selected. The included studies had high methodological quality. Recurrence free survival* (progression/recurrence/relapse/disease/tumor-free survival were combined and redefined as RFS*) and overall survival (OS) outcomes were highest in MIS-treated patients (HR 0·57, 95% confidence intervals [CI] 0·38–0·85; HR 0.48,95% CI 0.36–0.64, respectively), followed by SR-treated patients (HR 0.60, 95% CI 0.50–0.74; HR 0.62, 95% CI 0.55–0.72, respectively). TACE was highly efficacious (58.9%) at decreasing the rates of major complications. Similar findings were obtained through sensitivity analysis, and in most of the prognostic subgroups.

**Conclusions:**

MIS and SR exhibited the highest clinical efficacies, however, they were associated with higher rates of complications. Ablation is effective in small tumors, whereas SBRT is a relatively promising treatment option for SHCC. More well-designed, large-scale randomized controlled trials should be performed to validate our findings.

**Supplementary Information:**

The online version contains supplementary material available at 10.1186/s12935-021-02365-1.

## Introduction

Globally, hepatocellular carcinoma (HCC) is the sixth most prevalent tumor and the fourth leading cause of cancer-related deaths [1]. Due to the increase in HCC-related mortality, studies are evaluating optimal therapeutic options for this cancer [2, 3]. Cancer surveillance has resulted in early tumor detection, thereby improving the treatment outcomes for very early or early stage HCC, also known as small HCC (SHCC).

The most common therapeutic strategies for SHCC include surgery (such as liver transplantation or surgical resection (SR)), ablation (such as radiofrequency ablation (RFA)), microwave ablation (MWA), cryotherapy ablation (CRA), percutaneous ethanol injection (PEI), non-catheter based therapies, such as stereotactic body radiotherapy (SBRT) and catheter based embolic therapies such as transarterial chemoembolization (TACE). Liver transplantation is the most effective treatment option for SHCC (up to 75% to 92% 5-year survival rate) [4]. However, liver transplantation is limited by high costs of the procedure and organ shortage [5]. The European Association for the Study of the Liver (EASL) [6] and American Association for the Study of Liver Diseases (AASLD) [7] recommend surgical resection as the first-line treatment option for SHCC. However, ablation is an effective alternative for patients that are not eligible for surgery. The recently developed minimally invasive liver surgery (MIS) strategy is a safe and effective approach for liver resection [8–10]. Compared to traditional surgery, MIS has a significant short-term efficacy advantage and a similar long-term efficacy [11]. However, the choice of MIS or traditional surgery is challenging. The possibility of complete tumor resection at the early stages has led to the development of several treatment options, including RFA, MWA, PEI and CRA [12–14].

Ablation induces the necrosis of neoplastic cells by modifying the local temperature. This strategy is associated with several advantages, including minimal invasiveness, high safety, cost-effectiveness, and reproducibility. RFA is the most common ablative technique and, in selected patients, it has been shown to exhibit comparable efficacies to surgery. It is an effective replacement therapy for SHCC [15]. In recent years, various ablation methods, such as MWA and CRA have been widely used [16–18]. MWA is a local ablation modality [19] that uses a similar technology as radiofrequency ablation. However, MWA is characterized by higher thermal efficiencies and it requires less ablation time. Compared to RFA, MWA is less susceptible to large vessels that are adjacent (the heat sink effect) to the tumor and is more effective for larger tumors (3–4 cm in size) [20–22]. In addition, CRA has a comparable efficacy to RFA. Occasionally, CRA is used in high-resource settings [23–25]. When tumor nodules are near large intrahepatic blood vessels or bile ducts, PEI is the preferred treatment method to avoid thermal potential damage by RFA or MWA to these organs [26]. SBRT [27] is an emerging local modality with potent local control rates of 91% for tumors less than 5 cm and 74% for tumors ≥ 5 cm in size [28]. Compared to best supportive care, TACE is associated with significantly longer overall survival outcomes, [29]. TACE involves intravenous infusions of cytotoxic chemotherapeutic agents. The delivery of embolization particles into the feeding artery of tumors leads to ischemic necrosis of the tumor [30]. Although TACE has a high efficacy, assessment of its effectiveness is challenging. This is because TACE refers to a wide variety of interventions with variable end-points [31].

Therefore, there is no consensus on optimal treatment options for very early or early-stage HCC. In cases where large clinical trials with multiple comparator arms are not available, bayesian network meta-analysis can be used to compare different treatment methods to identify the most effective approach [32]. A random effects network meta-analysis was conducted to compare the efficacies and safety of the primary therapeutic options of SHCC, thereby establishing an optimal treatment for very early or early-stage HCC. Similar studies have been conducted. However, this study included the latest treatments and latest studies. In addition, HR, which is the most reliable effect indicator in survival analyses, relative to RR and OR, was used in comparisons.

## Methods

### Search strategy

All procedures in this meta-analysis were performed in accordance with PRISMA (Preferred Reporting Items for Systematic Reviews and Meta-Analyses) guidelines [33]. Relevant studies published between January 2010 and February 2021 were searched in EMBASE, PubMed, Cochrane Library, Web of science, and conference proceedings. Clinical management of hepatocellular carcinoma has improved in the past 10 years, therefore, relevant studies published from 2010 were included in this study. Searches were conducted using various combinations of Medical Subject Headings (MeSH) and non-MeSH terms. Manual searches were conducted for relevant studies identified from the bibliographies of retrieved articles.

### Eligibility criteria

Eligibility criteria included the study population, intervention, comparison, outcome, and study design (PICOS) [34]. i. The study population comprised very early and early HCC (defined as single nodule < 5 cm in diameter or up to 3 nodules with the diameter of each nodule being < 3 cm) patients. ii. Studies that compared at least 2 intervention techniques, including: RFA, MWA, SR (surgical resection or liver transplantation), TACE, PEI, MIS, SBRT, or CRA. iii. Studies reporting on various outcomes, including OS, RFS, PFS, DFS, TFS or major complication rates and iv. Studies that used RCTs or Non-RCTs study designs.

The exclusion criteria were: i. Case reports, letters to the editor, editorials and reviews were excluded; ii. Studies that focused on large HCC, intrahepatic recurrent small HCC, small HCC with extrahepatic metastases or vascular invasions, as well as those that focused on Child–Pugh classification of C or above; iii. Studies that did not report the relevant outcomes, and iv. Studies whose reported data were replicated in already included studies were excluded.

### Study selection and data extraction

Duplicates were excluded and titles as well as abstracts of the retrieved articles independently screened by two investigators (SY and HPL) using Endnote 7X (Clarivate Analytics; Philadelphia, PA, USA) to determine if they met the inclusion criteria. Full texts of the selected articles were reviewed to determine if they were eligible for inclusion in the analysis. Two authors (SY and JNS) extracted and summarized the data from included studies, including first author names, publication dates, study settings, study designs, mean duration of follow-up, general characteristics, disease characteristics, OS, RFS, DFS and major complication rates. Any disagreements were resolved by consultations with senior authors.

### Analysis of methodological qualities of the included studies

The quality of non-randomized trials was independently evaluated by two investigators using the Newcastle Ottawa Scale [35]. Each study was assigned a maximum of nine stars (six or more stars were considered high quality). The quality of RCTs was determined using the Cochrane’ s Risk of Bias Tool for randomized trials, which comprises seven specified domains [36]. Then, RCTs were classified into three categories: low risk, high risk, and having some concerns. Two reviewers (SY and HPL) independently assessed the quality of the included studies. Disagreements were settled by discussion or by consulting a third reviewer.

### Statistical analysis

In this meta-analysis, RFS, PFS, DFS and TFS were combined and redefined as RFS*. DFS was the time from randomization to tumor recurrence or death. PFS was the time between randomization and death or progression (Time to progression is a related, less-preferred end point wherein deaths without progression are censored observations rather than being counted as events) [37]. TFS was the time from randomization to metastasis or recurrence [38]. These terms are not synonyms, but can sometimes represent the same outcome. OS was the time from randomization to the time of all-cause death [39].

Network meta-analysis (NMA) was performed using natural log transformations of Hazard ratios (HRs) and their 95% confidence intervals (CIs) to estimate standard errors (SEs), which consider the number and time of events. Hazard ratios (HRs) with 95% CI were used to determine effect sizes for OS, RFS and DFS. Odds ratios (ORs) with 95% CI were calculated to determine the effect sizes of major complication rates.

A network-node plot of comparisons was generated to indicate the number of trials that formed direct comparisons between treatment groups. RFA was used as the common parameter for comparisons in order to include all trials within 1 framework. It was assumed that efficacy would not vary based on dosages or schemes. Results were validated using “gemtc” (version 0.8–8) in R (version 4.0.3) and JAGS (version 4.3.0) softwares with identical parameter settings. A random-effects consistency model was used for each outcome measure. Three independent Markov chains were established for running 100 000 interactions with 10 000 burn-in samples and 10 thinning rates. The process was conducted to obtain a posterior distribution. Model convergence of iterations was evaluated and visualized using trace plots and Brooks-Gelman-Rubin diagnostics. Global inconsistencies were not present, therefore, NMA was performed following the consistency framework [40]. Node-splitting models were used to assess local consistency, and to test whether the results from direct and indirect comparisons were consistent within treatment loops [41]. Statistical heterogeneity and overall network consistency were determined using Q test and statistic inconsistency index (I2). An I^2^ value > 50% indicated a significant level of heterogeneity, therefore, sensitivity analyses were conducted by omitting one study at a time to identify heterogeneity sources [42]. Multivariate data were directly extracted from studies. Univariate HR data were extracted if multivariate data were not available. For studies that did not report HR values, the Engauge Digitizer software (version 4.1, M Mitchell) was used to extract data from Kaplan–Meier plots.

## Results

### Study characteristics

**Fig. 1:**
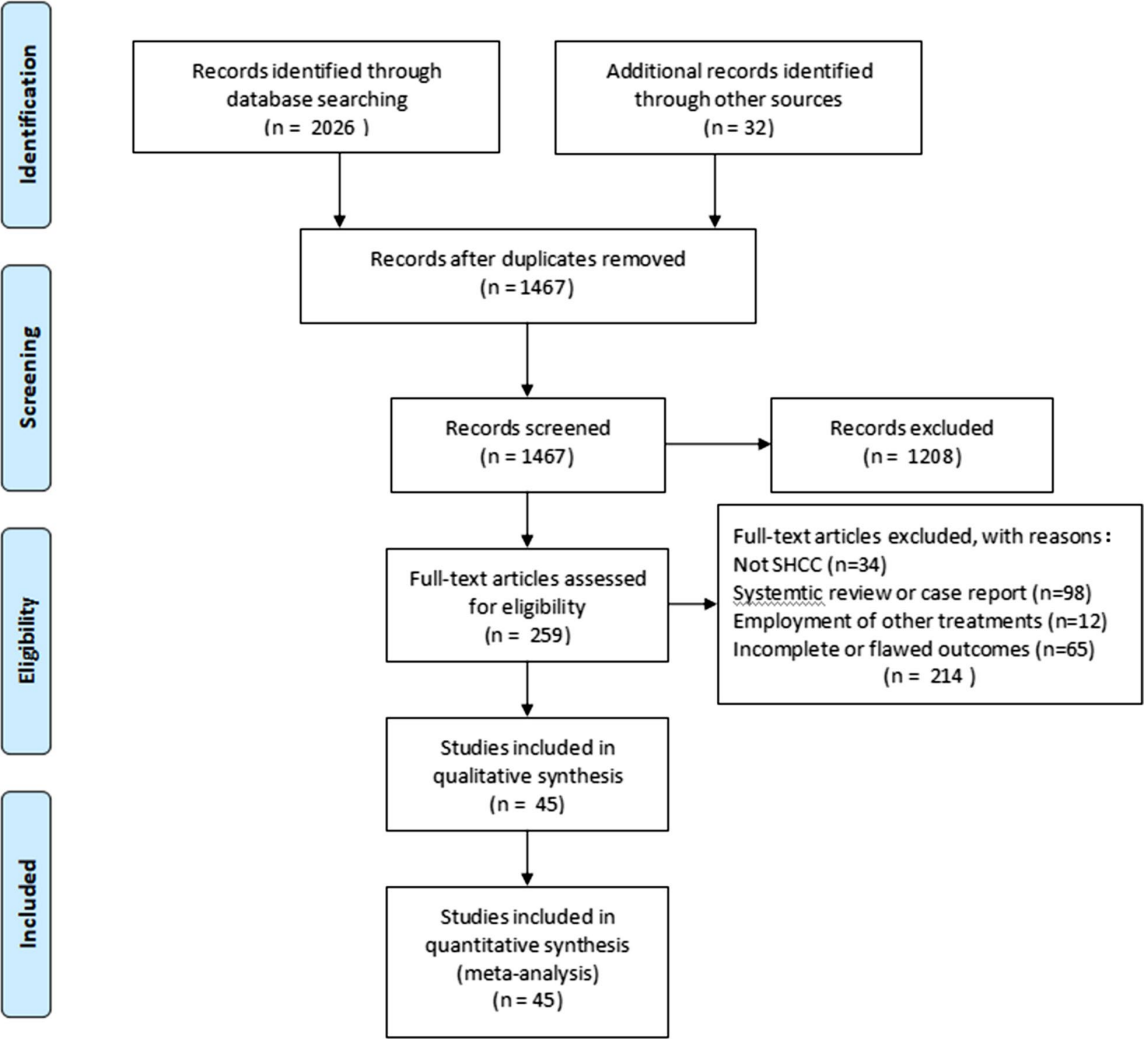
Preferred Reporting Items for Systematic Reviews and Meta-Analyses (PRISMA) flow diagram showing literature search and selection processes

A total of 2058 potentially relevant articles were identified from database searches. Then, after removal of duplicates at the initial stage of title and abstract reviews, 1208 articles were excluded because they did not meet the inclusion criteria. Full-texts for 259 articles were retrieved for detailed reviews and assessments. Notably, a total of 214 records were excluded, and 45 articles [20, 43–85] involving 11,364 patients were included in the final analysis. All included studies reported on overall survival outcomes for patients. Of the 45 included studies, 10 were RCTS while 35 were non-randomized intervention studies (Fig. 1). Two trials were three-arm studies, one comparing SR, TACE, and RFA, the other comparing RFA, MIS and SR, whereas the other trials were two-arm trials. Study characteristics of the RCTs and non-randomized studies are presented in Tables 1 and 2.

**Table 1:** Characteristics of included randomized controlled trials.

Author (No.)	Year	Country	Treatment	Sample size	Mean ± SD/Median (range) age, year	Sex (male/female)	Child A/B	Mean ± SD/Median (range) size, cm	follow up (months)	Outcomes
Vietti Violi N (1)	2018	Switerland	MWA	71	68(60–72)	59/12	57/14	1.8 ± 0.65	36	OS, RFS, C
			RFA	73	65(59–73)	6/11	53/20	1.8 ± 0.71		
Yu J (2)	2017	China	MWA	203	NR	NR	NR	2.7(0.7–5.0)	81.9	OS, DFS, C
			RFA	200				2.6(0.9–5.0)		
Kamal A (3)	2019	Egypt	MWA	28	55(42–80)	21/7	22/6	3.25(2.0–5.0)	12	RFS, C
			RFA	28		22/6	22/6	3.28(1.7–4.5)		
Abdelaziz A (4)	2014	Egypt	MWA	66	53.6	48/18	25/41	2.90 ± 0.97	40	OS, C
			RFA	45	56.8	31/14	24/21	2.95 ± 1.03		
Feng K (14)	2012	China	SR	84	47(18–76)	75/9	43/41	2.6 ± 0.8	36	OS, RFS, C
			RFA	84	51(24–83)	79/5	39/45	2.4 ± 0.6		
Fang Y (15)	2014	China	SR	60	53.5 ± 11.0	46/18	43/17	2.3 ± 0.4	40	OS, DFS, C
			RFA	60	51.4 ± 8.1	42/18	32/23	2.2 ± 0.5		
Ng KKC (24)	2017	China	SR	109	55(31–82)	89/20	107/2	2.9(1.0–5.0)	120	OS, DFS, C
			RFA	109	57(23–78)	86/23	104/5	2.6(1.0–5.0)		
Lee HW (25)	2018	Korea	SR	29	55.6 ± 7.9	23/6	29/0	≤4	93.6	OS, DFS, C
			RFA	34	56.1 ± 7.4	24/10	31/3			
Giorgio A (30)	2011	Italy	PEI	143	62(44–78)	102/41	75/68	2.27(1.3–2.9)	68	OS, C
			SR	142		105/37	70/72	2.34(1.1–3.0)		
Wang C (43)	2015	China	CRA	180	53.9 ± 9.6	140/40	121/59	≤4	64	OS, RFS, C
			RFA	180	53.3 ± 8.9	150/30	109/71		65	

*NR* Not report, *OS* Overall Survival, *RFS* Recurrence/ Relapse-free survival, *DFS* Disease-free survival, *C* major complications rate

**Table 2:** Characteristics of included Non-randomized controlled trials

Author (No.)	Year	Country	Treatment	Sample size	Mean ± SD/Median (range) age, year	Sex (male/female)	Child A/B	Mean ± SD/Median (range) size, cm	follow up (months)	NOS	Outcomes
Du S (5)	2020	China	MWA	218	56.4 ± 10.3	173/45	200/18	2.9 ± 1.2	51	9	OS, RFS, C
			RFA	234	57.3 ± 9.3	192/42	216/18	2.4 ± 1.0			
Cillo U (6)	2014	Italy	MWA	42	64 (47–81)	35/7	NR	2.5(1.5–5.3)	24	9	OS
			RFA	100	63 (34–81)	83/17		3.0(1.0–6.0)			
Ghweil A (7)	2019	Egypt	MWA	25	NR	18/7	NR	≤ 5	48	8	OS, DFS, C
			RFA	30		21/9					
Zhang L (8)	2013	China	MWA	77	54 ± 9.5 (26–76)	67/10	77/0	≤ 5	70	8	OS, DFS, C
			RFA	78	54 ± 10.5 (30–80)	64/4	78/0				
Vogl TJ (9)	2015	Egypt	MWA	28	60 (45–68)	23/5	NR	3.6 (0.9–5.0)	42	8	OS, C
			RFA	25	57 (40–64)	19/6		3.2 (0.8–4.5)			
Potretzke TA(10)	2016	USA	MWA	99	61 (44–82)	81/18	NR	2.2 (2.0–2.3)	48	7	OS, TFS, C
			RFA	55	62 (23–88)	40/15		2.4 (2.2–2.6)			
Ding J (11)	2013	China	MWA	113	59.06 ± 11.68 (30–86)	85/28	75/38	2.6 ± 0.9 (0.8–5.0)	60	7	OS, DFS, C
			RFA	85	58.64 ± 8.52 (40–77)	68/17	49/36	2.4 ± 0.8 (1.0–4.8)			
Xu Y (12)	2017	China	MWA	301	54.2 ± 11.0	235/23	278/23	1.7 ± 0.3	102	7	OS, RFS, C
			RFA	159	54.0 ± 11.0	132/27	140/19	1.7 ± 0.3			
Santambrogio R (13)	2017	Italy	MWA	60	70 ± 8.3 (66–76)	43/17	60/0	2.15 ± 0.53 (1.75–2.5)	45.8	8	OS, DFS, C
			RFA	94	69 ± 9(65–76)	69/25	94/0	1.92 ± 0.5 (1.5–2.2)			
Hung HH (16)	2011	China	SR	229	60.1 ± 12.6	184/45	NR	2.88 ± 1.06	60	8	OS
			RFA	190	67.4 ± 11.5	121/69		2.37 ± 0.92			
Wong KM (17)	2013	China	SR	46	55.1 ± 12	30/16	46/0	2.1 ± 0.6	60	8	DFS, C
			RFA	36	63.5 ± 13	18/18	36/0	1.9 ± 0.6			
Wang JH (18)	2012	China	SR	208	NR	168/40	205/3	≤ 5	NR	8	OS, DFS
			RFA	254		161/93	191/63				
Imai K (19)	2013	Japan	SR	101	63.3 ± 9.7	75/26	97/4	2.14 ± 0.55	49	8	OS, DFS
			RFA	82	67.6 ± 8.5	46/36	60/22	1.87 ± 0.50			
Yang HJ (20)	2014	Korea	SR	52	55.7 ± 10.6	38/4	50/2	≤3	96	8	OS, C
			TACE	66	59 ± 9.5	49/17	55/11				
			RFA	79	57.2 ± 9.2	59/20	68/11				
Hocquelet A (21)	2015	France	SR	103	68(61–74)	82/21	99/4	3.5 (2.5–4)	122	8	OS, C
			RFA	178	65(56–74)	148/30	145/33	2.2 (2–2.8)			
Liu PH (22)	2016	China	SR	109	60 ± 13	78/31	NR	2.6 ± 1.3	96	8	OS, RFS
			RFA	128	64 ± 12	84/44		2.0 ± 1.1			
Guan TP (23)	2017	China	SR	92	52.48 ± 8.36	75/17	79/13	3.19 ± 0.98	60	8	OS, RFS, C
			RFA	102	54.02 ± 7.66	90/12	83/19	3.10 ± 0.88			
Santambrogioa R (26)	2015	Italy	SR	76	66 ± 9(61–72)	52/24	79/13	1.75 ± 0.28 (1.5–2)	197	8	OS, C
			RFA	76	68 ± 8(63–73)	59/17	83/19	1.66 ± 0.36 (1.5–2)			
Oh JH (27)	2020	Korea	SR	48	54.5(50.2–61.7)	37/11	NR	≤ 3	84	8	OS, RFS, C
			TACE	141	62.0(54.5–67)	112/29					
			RFA	87	59(51.0–68.0)	72/15					
Hsiao CY (28)	2020	China	SR	156	58.8 ± 1.7(27–82)	95/61	NR	1.46 ± 0.31 (0.5–1.9)	65.1	7	OS, RFS
			RFA	231	62.2 ± 12.3(31–84)	141/90		1.58 ± 0.24 (0.8–1.9)			
Liang B (29)	2018	China	SR	64	48.8(26–74)	54/10	58/6	3.1 (1.5–5.0)	66	7	OS, RFS, C
			RFA	58	51.4(30–85)	48/10	53/5	2.9 (1.2–5.0)			
Di Sandro S (31)	2019	Italy	MIS	51	68(62–76)	33/17	NR	2.5 (2.0–3.0)	60	8	OS, RFS, C
			SR	341	66(59–73)	68/23		3.5 (2.5–5.6)			
			RFA	143	65(57–75)	66/25		2.0 (1.6–2.5)			
Santambrogio R (32)	2018	Italy	MIS	59	68 ± 9	42/17	59/0	2.09 ± 0.67 (1.5–2.6)	54	8	OS, C
			RFA	205	69 ± 9	152/53	205/0	1.91 ± 0.58 (1.5–2.2)			
Casaccia M (33)	2017	Italy	MIS	24	63.58 ± 9.55	16/8	22/2	3.3 ± 1.4	72	8	OS, DFS
			RFA	22	60.82 ± 7.25	18/4	12/10	2.6 ± 1.3			
Song J (34)	2015	China	MIS	78	48(44–57)	70/8	78/0	≤ 4	96	9	OS, RFS, C
			RFA	78	48(43–58)	70/8	78/0				
Vitali GC (35)	2015	Switerland	MIS	45	61.4(31–84)	30/15	40/5	2.3 (1–3)	129	8	OS, C
			RFA	60	67.3(47–83)	52/8	15/15	2.1 (2.1–3)			
Ogiso S (36)	2020	Japan	MIS	85	69(46–88)	62/23	73/12	2.1 (0.8–3)	153	8	OS, RFS, C
			RFA	136	73(47–87)	98/38	110/26	1.6 (0.5–3)			
Chong CC (37)	2020	China	MIS	59	57.7 ± 10.5	46/13	59/0	2.0 (1.6–2.8)	72.3	8	OS, DFS, C
			RFA	155	62.1 ± 9.8	120/35	143/2	2.0 (1.6–2.7)			
Wahl DR (38)	2016	USA	SBRT	63	62(35–85)	54/9	43/20	≤ 5	112.8	8	OS, C
			RFA	161	60(31–81)	117/44	80/81				
Kim N (39)	2019	Korea	SBRT	105	63.0 (35.0–86.0)	86/19	97/8	2.4 (0.7–5.5)	37.3	9	PFS, C
			RFA	668	64.0 (26.0–86.0)	523/145	599/69	1.6 (0.5–4.6)			
Sun J (40)	2020	China	SBRT	122	54.31 ± 9.35	90/32	111/11	2.62 ± 1.09	117	8	OS, RFS, C
			SR	195	52.20 ± 9.54	156/39	193/2	2.81 ± 1.13			
Nakano R (41)	2018	Japan	SBRT	27	72.6 ± 9.1	15/12	238/16	18.3 ± 1.4	60	8	OS, DFS, C
			SR	254	68.1 ± 10.2	183/71	23/4	19.4 ± 6.0			
Martin AN (42)	2019	USA	TACE	27	66.6 ± 8.7	19/8	NR	18 (13–27)	62.4	8	OS
			RFA	27	67.1 ± 11	15/12		20 (14–23)			
Xu J (44)	2018	USA	CRA	94	65.9±10.1	73/21	NR	28 (21–38)	120	7	OS
			RFA	3145	62.5±9.7	2353/792		27 (20–36)			
Hu J (45)	2019	China	CRA	56	54.9±11.3(28–75)	42/14	46/10	36.5 ± 8.5 (16–57)	46.2	8	OS, RFS, C
			WMA	64	55.2±7.2(32–73)	46/18	50/14	32.5±7.2 (16–42)			

*NR* Not report, *OS*: overall Survival, *RFS* recurrence/ relapse-free survival, *DFS* disease-free survival, *TFS* tumor-free survival, *PFS* progression-free survival, *C* major complications rate

The included studies showed a high methodological quality. Analysis using the Cochrane Collaboration tool showed a low risk of bias for the 10 randomized trials. The 35 non-randomized studies were of high quality (≥ 7/9 points on the Newcastle–Ottawa scale). Details on quality assessments of randomized and non-randomized studies are presented in Additional file 1: Fig. S1 and Table 2, respectively. A comparison-adjusted funnel plot for the eight therapies network was generated to determine the publication bias. There was no evidence of asymmetry (Additional file 2: Fig. S2).

### Network meta-analysis

**Fig. 2:**
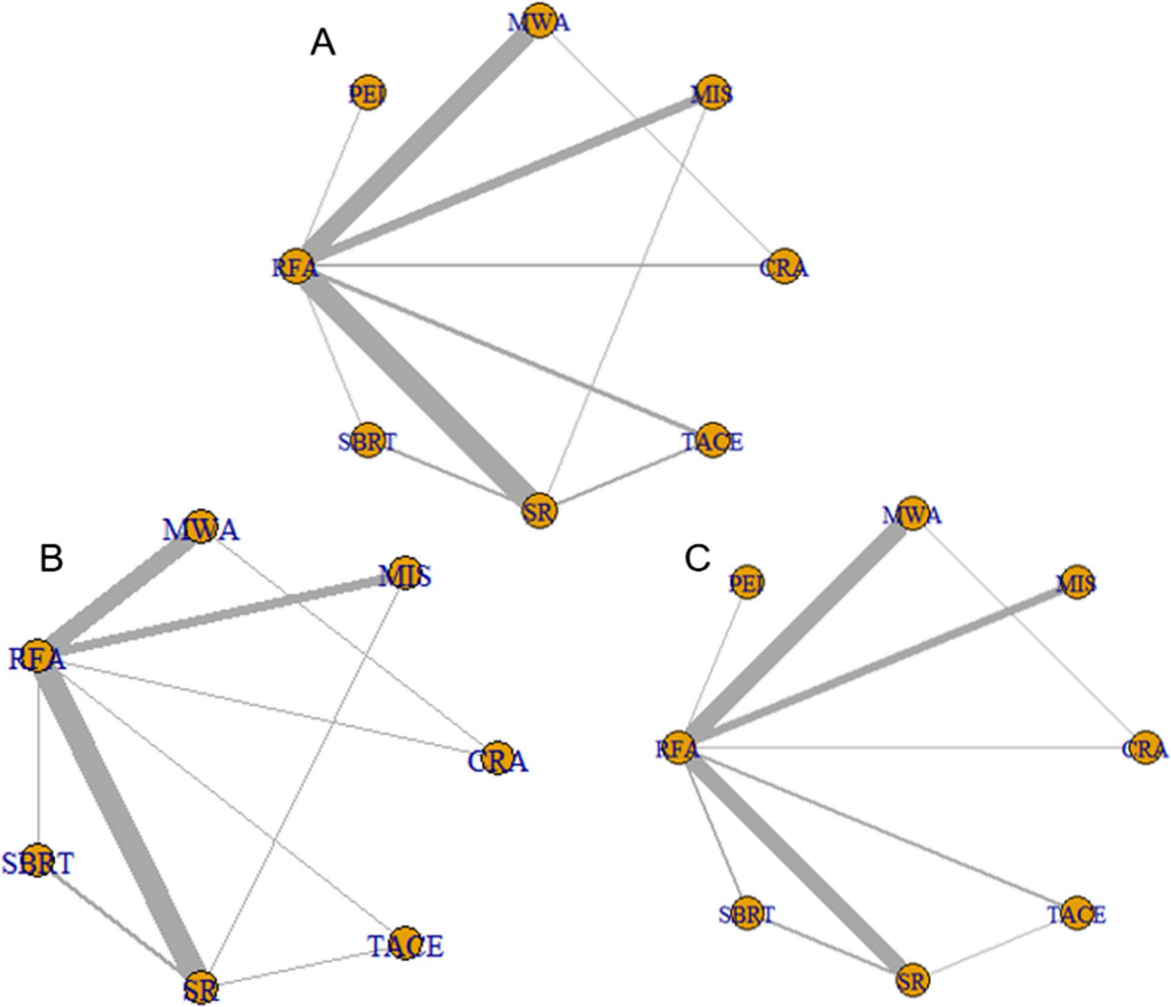
Network plots for included studies. **A** OS, **B** RFS* (RFS, PFS, DFS and TFS were combined and redefined as RFS*). **C** Major complications rate. *OS* Overall Survival, *RFS* recurrence-free survival, *PFS* progression-free survival, *DFS* disease free survival, *TFS* tumor-free survival

The NMA of interventional techniques for very early or early-stage HCC was conducted using the R-software (Fig. 2). The results are presented in the following subsections.

### Survival analysis

**Fig. 3:**
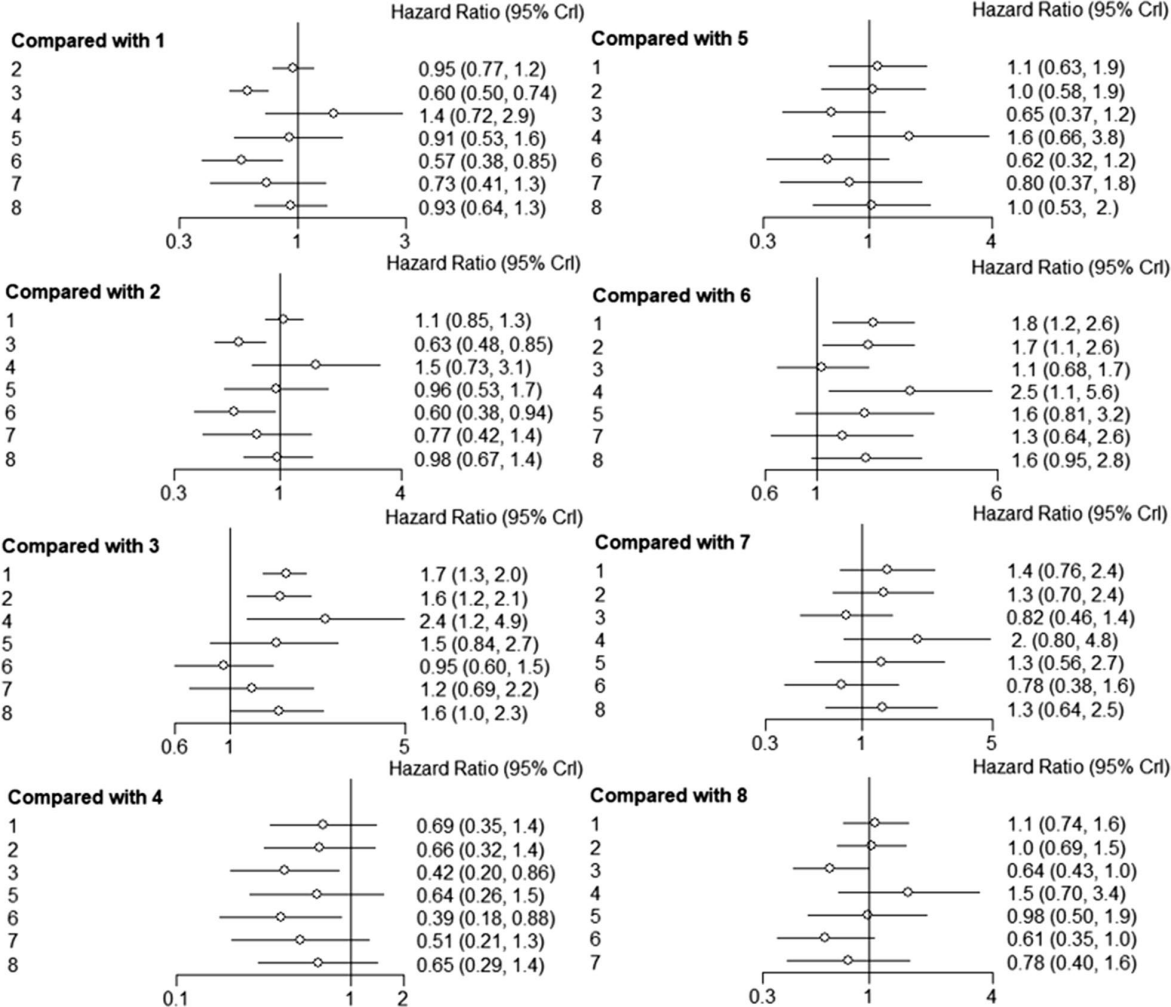
Forest plots showing the relationship between different interventional methods and OS of HCC patients HR values and 95% CI were used for comparisons. 1: RFA, 2: MWA, 3: SR; 4: TACE, 5: PEI, 6: MIS, 7: SBRT, 8: CRA. *OS* Overall Survival, *HCC* Hepatocellular Carcinoma, *HR* Hazard ratio, *CI* confidence interval, *RFA* radiofrequency ablation, *MWA* microwave ablation, *SR* surgical resection, *TACE* transarterial chemoembolization, *PEI* percutaneous ethanol injection, *MIS* Minimally invasive liver surgery, *SBRT* stereotactic body radiotherapy, *CRA* cryotherapy ablation

OS was reported in 43 trials. The HR and the corresponding 95% CI of OS were calculated after different treatments. Pooling of HRs for OS revealed a significant advantage for surgery, including SR and MIS, compared to RFA in network meta-analysis (HR 0.60, 95% CI 0.50–0.74 and HR 0.57, 95% CI 0.38–0.85, respectively). Pooling of HRs for OS showed a statistically significant advantage for SR, compared to MWA (HR 0.63, 95% CI 0.48–0.85), TACE (HR 0.42, 95% CI 0.20–0.86) and RFA (HR 0.60, 95% CI 0.50–0.74). Pooling of HRs for OS showed a statistically significant advantage for MIS compared to MWA (HR 0.60, 95% CI 0.38–0.94), TACE (HR 0.39, 95% CI 0.18–0.88) and RFA (HR 0.57, 95% CI 0.38–0.85). Analysis of OS for patients subjected to ablative electrochemical therapies and non-ablative treatment revealed a high efficacy for MWA, PEI, CRA and SBRT, while the effectiveness of TACE was low, relative to that of RFA. However, differences in OS outcomes after treatment with these therapeutic approaches were not significant. Compared to SR, MIS was associated with better OS outcomes (HR 0.95, 95% CI 0.60–1.5), however differences were not significant (Fig. 3).

**Fig. 4:**
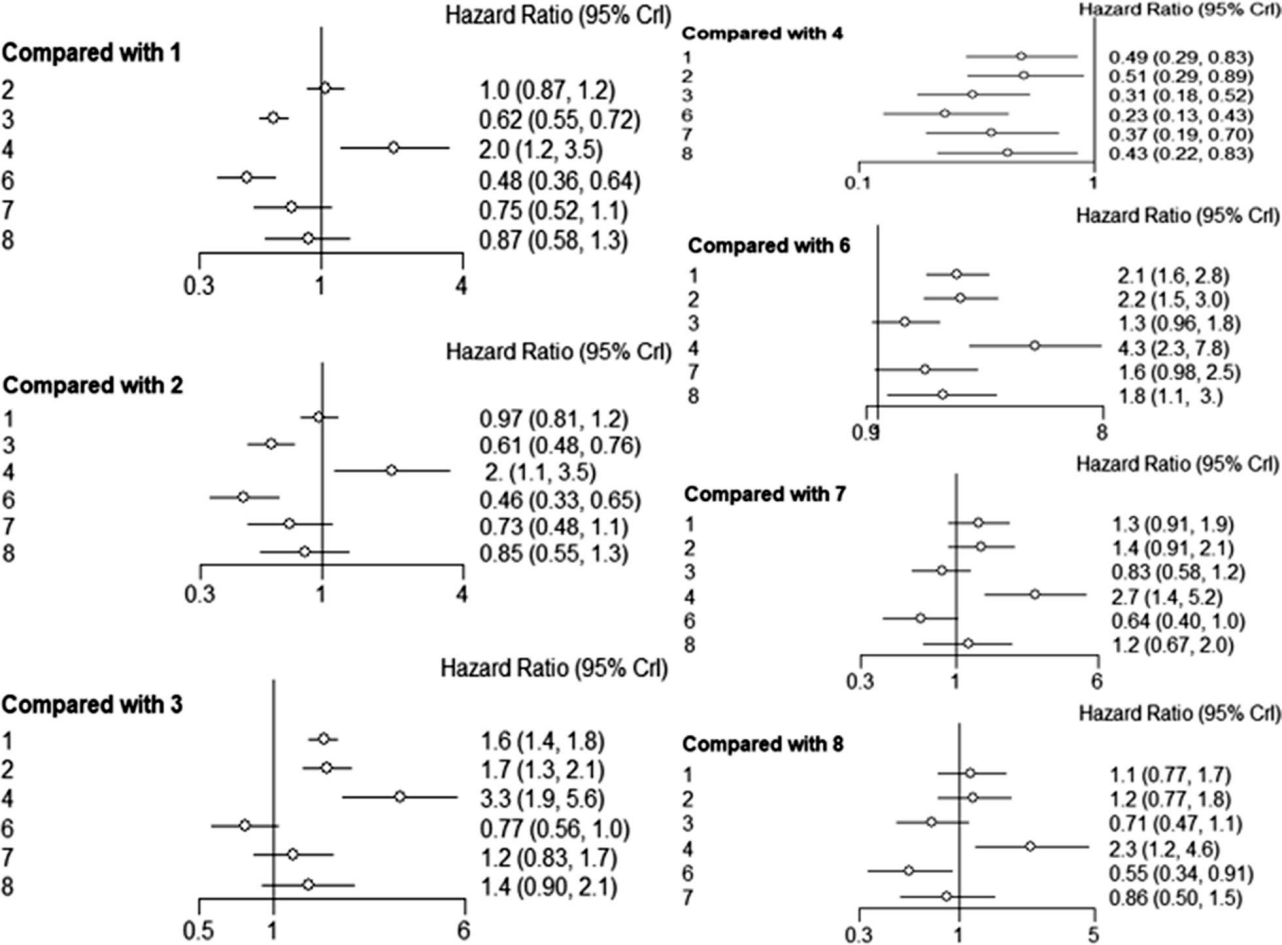
Forest plots showing the relationships between different interventional methods and RFS*. Comparisons were conducted using HR values and 95% CI. 1: RFA, 2: MWA, 3: SR; 4: TACE, 6: MIS, 7: SBRT, 8: CRA. *HR* Hazard ratio, *CI* confidence interval, *RFA* radiofrequency ablation, *MWA* microwave ablation, *SR* surgical resection, *TACE* transarterial chemoembolization, *MIS* Minimally invasive liver surgery, *SBRT* stereotactic body radiotherapy, *CRA* cryotherapy ablation

RFS* was reported in 32 trials comprising 7 treatments in addition to PEI. SR and MIS had a HR of 0.62 (95% CI 0.55–0.72) and 0.48 (95% CI 0.36–0.64), respectively, indicating a significant advantage compared to RFA. It was established that SBRT and CRA had better efficacies, whereas TACE was associated with poor RFS*, compared to RFA, however, the differences were not significant. Notably, RFA and MWA exhibited the same RFS. Pooling of HRs for RFS* revealed a significant advantage for SR, compared to MWA (HR 0.61, 95% CI 0.48–0.76) and TACE (HR 0.31, 95% CI 0.18–0.52). In addition, pooling of HRs for RFS revealed a statistically significant advantage for MIS, compared to MWA (HR 0.46, 95% CI 0.33–0.65), TACE (HR 0.23, 95% CI 0.13–0.43) and CRA (HR 0.55, 95% CI 0.34–0.91). All other treatments exhibited significant advantages, relative to TACE (Fig. 4).

**Fig. 5:**
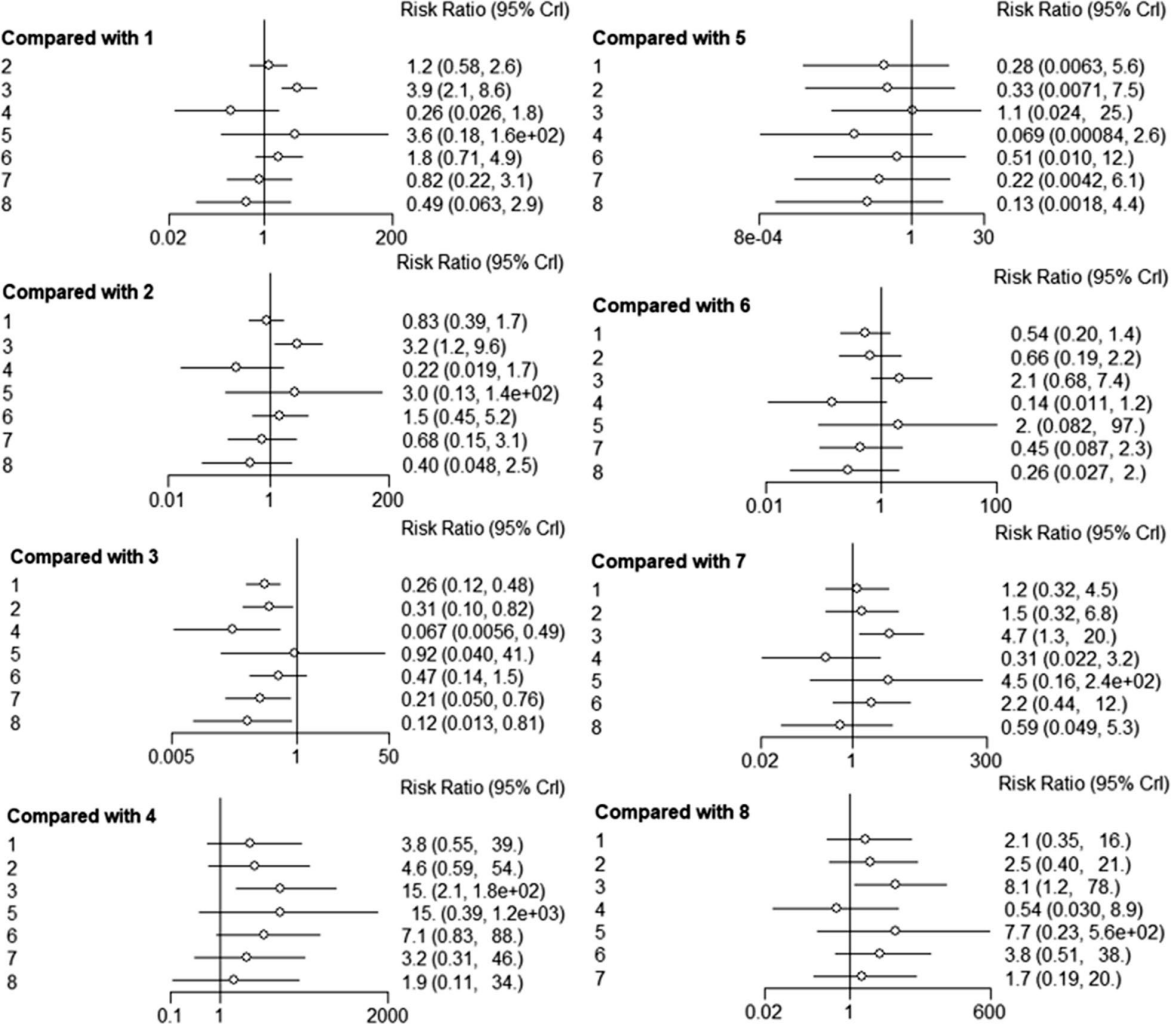
Forest plots showing the relationships between different interventional methods and major complication rates, compared using RR values and 95% CI. 1: RFA, 2: MWA, 3: SR; 4: TACE, 5: PEI, 6: MIS, 7: SBRT, 8: CRA. *RR* risk ratio, *RFA* radiofrequency ablation, *MWA* microwave ablation, *SR* surgical resection, *TACE* transarterial chemoembolization, *PEI* percutaneous ethanol injection, *MIS* Minimally invasive liver surgery, *SBRT* stereotactic body radiotherapy, *CRA* cryotherapy ablation

Bayesian network meta-analysis indicated that SR (RR 2.70, 95% CI 1.70–4.30; RR 2.42, 95% CI 1.23–4.74, respectively) was significantly associated with more severe complications, compared to RFA- and MWA-associated complications. Moreover, TACE (RR 0.14, 95% CI 0.03–0.60) and SBRT (RR 0.23, 95% CI 0.08–0.67) were associated with significantly less severe complications, compared to TACE. Furthermore, pooled RR revealed a significant advantage for TACE, compared to MWA (RR 4.78, 95% CI 1.06–21.47) (Fig. 5).

### Consistency analysis

Adaptation simulation with 10,000 iterations was used for development of the final model for all three outcome parameters. Initial simulation results from MCMC analysis were excluded from the model. The thinning factor was maintained, and the number of chains was set at 4. The adequacy of convergence for Gelman Rubin diagnostics approached 1 for all outcome parameters. The developed model for model diagnostics is presented in Additional file 3: Fig. S3. Analysis of global inconsistency did not reveal a significant shift in DIC (difference < 5) between the consistency and inconsistency models, implying that the data was consistent. Based on empirical data, node splitting analysis did not show any local inconsistency (Additional file 16: Table S1).

### Ranking of treatments

**Fig. 6:**
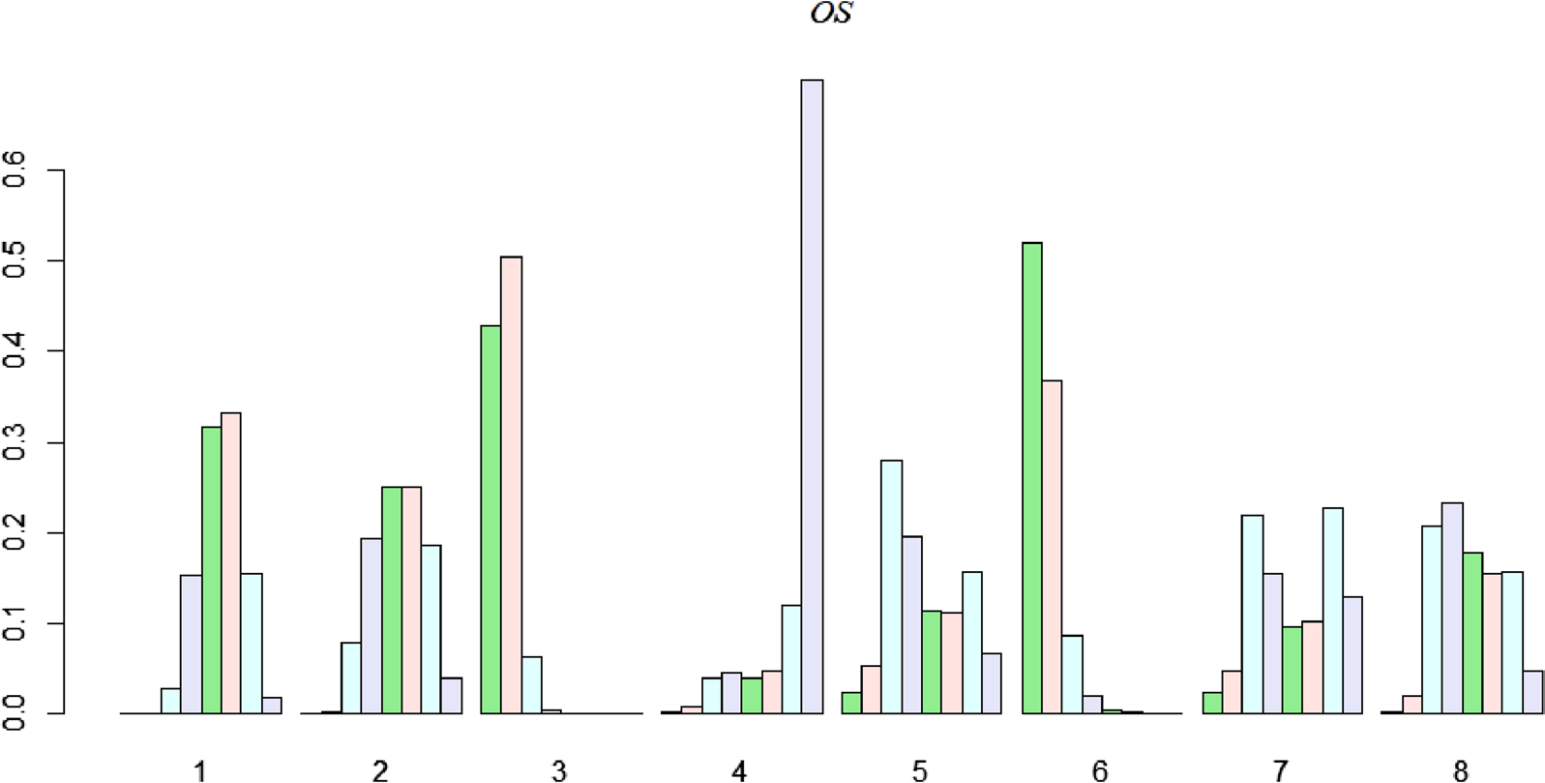
Efficacy levels of different treatment approaches based on OS. 1: RFA, 2: MWA, 3: SR, 4: TACE, 5: PEI, 6: MIS, 7: SBRT, 8: CRA. *OS* Overall Survival, *RFA* radiofrequency ablation, *MWA* microwave ablation, *SR* surgical resection, *TACE* transarterial chemoembolization, *PEI* percutaneous ethanol injection, *MIS* Minimally invasive liver surgery, *SBRT* stereotactic body radiotherapy, *CRA* cryotherapy ablation

**Fig. 7:**
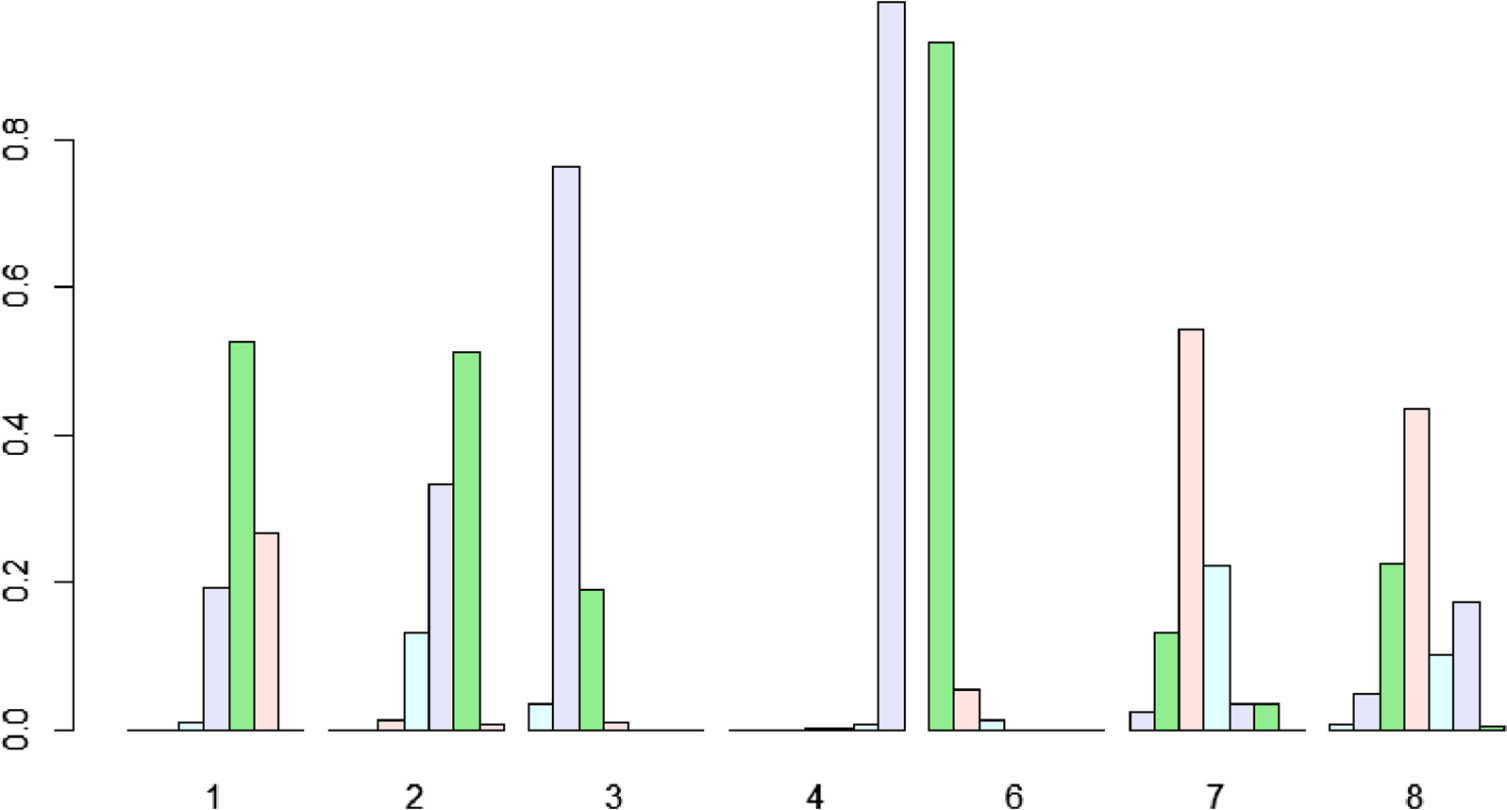
Efficacy levels of different treatment approaches based on RFS* (RFS, PFS, DFS and TFS were combined and redefined as RFS*). 1: RFA, 2: MWA, 3: SR, 4: TACE, 6: MIS, 7: SBRT, 8: CRA. *RFS* recurrence-free survival, *PFS* progression-free survival, *DFS* disease free survival, *TFS* tumor-free survival, *RFA* radiofrequency ablation, *MWA* microwave ablation, *SR* surgical resection, *TACE* transarterial chemoembolization, *MIS* Minimally invasive liver surgery, *SBRT* stereotactic body radiotherapy, *CRA* cryotherapy ablation

**Fig. 8:**
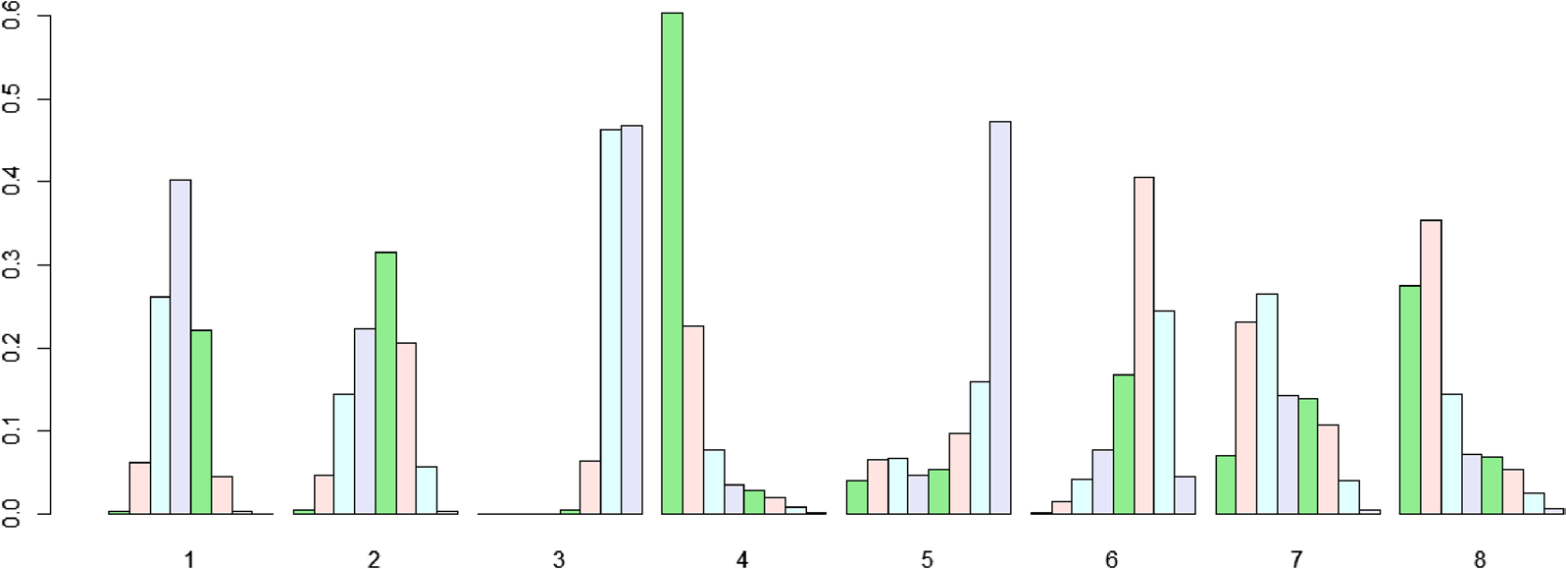
Efficacy levels of different treatment approaches based on major complication rates. 1: RFA, 2: MWA, 3: SR, 4: TACE, 5: PEI, 6: MIS, 7: SBRT, 8: CRA. *RFA* radiofrequency ablation, *MWA* microwave ablation, *SR* surgical resection, *TACE* transarterial chemoembolization, *PEI* percutaneous ethanol injection, *MIS* Minimally invasive liver surgery, *SBRT* stereotactic body radiotherapy, *CRA* cryotherapy ablation

According to the probability of being the optimal intervention based on associated OS values, each treatment was ranked at each of the possible eight positions (Fig. 6). Rank probability test indicated that MIS had the highest probability of being the optimal intervention (1 with 51.3%), SR was ranked second, SBRT was third, followed by PEI and CRA, MWA, while RFA was sixth. Notably, TACE was ranked as the worst possible intervention. Analyses of effectiveness according to increasing RFS* revealed consistent results as the results for overall survival outcomes (Fig. 7). MIS had a 89.9% probability of being the most optimal intervention, SR was ranked second, SBRT was third, CRA was fourth, followed by RFA and MWA, while TACE was the worst possible intervention. MIS and SR had the highest cumulative probabilities of improving OS and RFS outcomes, indicating that MIS and SR were the most effective treatments, compared to the other six interventions. TACE (58.9%) was ranked the most effective intervention in reduction of severe complication rates, SBRT was ranked second, followed by CRA, RFA, MWA, MIS and PEI, respectively, with SR being the worst possible intervention for reducing severe complications (Fig. 8).

### Subgroup analysis results

Subgroup analyses were performed based on tumor sizes (HCCs ≤ 3 cm and ≤ 5 cm) and study designs (RCT and Non-RCT) with RFS as the endpoint. Moreover, subgroup analyses were conducted according to tumor sizes (HCCs ≤ 3 cm and ≤ 5 cm) and study designs (RCT and Non-RCT) with OS as the endpoint. Studies that reported on OS outcomes were assigned into ≤ 3 cm (10 studies) and ≤ 5 cm (20 studies) subgroups. Pooled data showed significant benefits for SR, compared to RFA (HR: 0.59; 95% CI 0.43–0.85 or WMA (HR: 0.61; 95% CI 0.41–0.95) for HCC ≤ 5 cm (Additional file 4: Fig. S4). In addition, MIS exhibited a high efficacy for SR, compared to RFA (HR: 0.44; 95% CI 0.23–0.86) and MWA (HR: 0.46; 95% CI 0.23–0.93). However, SR and MIS did not exhibit a significant efficacy compared to RFA in patients with HCC ≤ 3 cm (Additional file 5: Fig. S5). RCTs provide high-level evidence using the most reliable methods in evaluating the most effective endpoints [86]. Rank orders for these treatments in relation to better OS were: SR > CRA > RFA > WMA > PEI in the RCTs subgroup (Additional file 6: Fig. S6). However, differences among these treatments were not significant. Notably, there was a potential selection bias in relatively low-level evidence in non-RCTs. MIS showed significantly high benefits (HR 0.55; 95% CI 0.36–0.84), compared to RFA and SR (HR 0.53; 95% CI 0.44–0.66) in the non-RCTs subgroup (Additional file 7: Fig. S7). In addition, MIS (HR 0.60; 95% CI 0.37–0.96) exhibited significantly more benefits with regards to OS outcomes, compared to WMA, SR (HR 0.75; 95% CI 0.43–0.78). SR and MIS exhibited the highest cumulative probabilities of being ranked first and second, respectively. Overall ranking of most treatments based on efficacy was comparable to ranking results of all subgroups. The main difference was that SBRT had the highest cumulative probabilities of being ranked the worst intervention in achieving maximum OS outcome benefits in the ≤ 3 cm subgroup, whereas SBRT was ranked third in the other subgroups.

Subgroup analyses for studies that included RFS as an endpoint showed that MIS and SR was associated with significantly better RFS in RFS and DFS subgroups, compared to RFA. Pooled data based on DFS associated with the five treatments showed that RFA and MWA were associated with lower DFS, compared to SR (HR 0.70, 95% CI 0.53–0.95; HR 0.65, 95% CI 0.42–0.99, respectively) and MIS (HR 0.43, 95% CI 0.22–0.85; HR 0.40, 95% CI 0.19–0.83, respectively) (Additional file 8: Fig. S8). Seventeen studies comprising 7 treatments other than PEI reported on RFS outcomes (Additional file 9: Fig. S9). Network meta-analysis showed that both SR and MIS were associated with significantly better RFS, compared to RFS outcomes associated with RFA (HR 0.55, 95% CI 0.50–0.63; HR 0.49, 95% CI 0.37–0.65, respectively), MWA (HR 0.58, 95% CI 0.46–0.74; HR 0.52, 95% CI 0.36–0.73, respectively), and CRA (HR 0.62, 95% CI 0.45–0.88; HR 0.55, 95% CI 0.35–0.94, respectively) treatments. In addition, SBRT was associated with better RFS outcomes, compared to RFA (HR 0.55, 95% CI 0.35–0.86) and WMA (HR 0.58 95% CI 0.35–0.94). A total 8 studies evaluated 4 treatments, excluding TACE, PEI, MIS, and SBRT in RCTs subgroup (Additional file 10: Fig. S10). There were no significant differences in RFS* rate among the treatments. A total of 24 studies in the non-RCTs subgroup investigated 7 treatments, with the exception of PEI (Additional file 11: Fig. S11). Pooled data showed that SR (HR 0.58, 95% CI 0.51–0.67; HR 0.55, 95% CI 0.43–0.68, respectively) and MIS (HR 0.48, 95% CI 0.36–0.63; HR 0.44, 95% CI 0.31–0.62, respectively) were associated with higher RFS outcomes, compared to RFA and WMA interventions. A total of 17 studies in the HCC ≤ 5 cm subgroup included 6 treatments and did not report TACE and PEI (Additional file 12: Fig. S12). Network meta-analysis showed that SR and MIS were associated with significantly high RFS, compared to RFA (HR 0.59, 95% CI 0.50–0.72; HR 0.41, 95% CI 0.25–0.70, respectively) and MWA (HR 0.59, 95% CI 0.46–0.75; HR 0.41, 95% CI 0.24–0.71, respectively) interventions. The HCC ≤ 3 cm subgroup (Additional file 13: Fig. S13) comprised 6 studies that reported findings on 4 treatments. Network meta-analysis showed that SR (HR 0.34, 95% CI 0.12–0.96) and MIS (HR 0.22, 95% CI 0.057–0.79) were associated with significantly higher RFS, compared to TACE intervention. Overall ranking of most treatments based on efficacy was similar to the ranking of all subgroups. The main difference was that MWA exhibited the highest cumulative probabilities of being ranked last in achieving maximum RFS benefits for the RCTs subgroup, whereas in most other subgroups MWA showed higher benefits, compared to some other treatments. Moreover, RFA was ranked above WMA in the RFS* group, whereas, WMA was ranked above RFA in other groups.

In addition, subgroup analyses were performed according to tumor sizes and study designs for severe complication rates. Analysis of studies in the RCT subgroup (Additional file 14: Fig. S14) involving 5 treatments (RFA, MWA, SR, PEI and CRA), showed that SR (RR 2.7, 95% CI 1.1–8.0) was the only intervention that was associated with significantly poor outcomes in terms of reducing tumor sizes, compared to RFA. MWA exhibited the highest cumulative probabilities of being ranked first in reducing tumor sizes, whereas SR was ranked last. Pooled data for the non-RCT subgroup (Additional file 15: Fig. S15) comprising studies reporting 7 treatments with the exception of PEI showed less severe complications for RFA, MWA, TACE and SBRT compared to SR (RR 0.23, 95% CI 0.085–0.51; RR 0.27, 95% CI 0.067–0.88; RR 0.062, 95% CI 0.0048–0.46; RR 0.21, 95% CI 0.05–0.71, respectively). CRA exhibited the highest cumulative probabilities of being ranked first in alleviation of complications, whereas SR was ranked last.

## Discussion

In this network meta-analysis, eight primary treatments for SHCC were compared through direct and indirect evidence reported in 45 studies involving 11,364 patients. Patients had tumor stages corresponding to BCLC 0 and BCLC A of the Barcelona Clinical Liver cancer (BCLC) staging system. Bayesian network meta-analysis showed that MIS and SR exhibited better OS and RFS outcomes, relative to the other non-surgical treatment methods. In addition, MIS was associated with better outcomes, compared to SR while SBRT was more effective at increasing RFS outcomes, relative to the other non-surgical treatment approaches, whereas TACE was associated with significantly poor RFS* outcomes, compared to the other six treatment methods. Subgroup analysis revealed that RFA was more effective in patients with small nodules (< 2 cm or 3 cm in diameter). Notably, tumor nodule sizes were the main causes of heterogeneity. Borderline observations were made between MIS and CRA for OS (HR 0.60, 95% CI 0.36–1.00), and between MIS and SR for RFS* (HR 0.77, 95% CI 0.56–1.00). Studies with larger sample sizes should be conducted to verify these findings. Findings from RCT and non-RCT subgroups were consistent with these findings. Subgroup analyses based on liver status (Child–Pugh score) [87], AFP, vascular invasion (the most important predictors of prognosis [88]), medical comorbidities and tumors sizes at 2 to 3 cm or 3 to 5 cm were not performed due to a lack of sufficient data. Well-designed, large-scale randomized controlled trials, including more subgroup analyses should be conducted. Ranking of the relative efficacy and safety for different treatment approaches provides a basis for making future clinical decisions for the most effective interventions for treatment of SHCC patients.

EORTC developed clinical practice guidelines that recommend SR and MIS as first line treatment options for SHCC [89, 90]. Surgical interventions involving the removal of the entire Couinaud segment containing tumors effectively eliminate the primary tumors. Therefore, cancer embolus and microscopic lesions are completely eliminated in patients who undergo surgery [91]. This may explain the relatively higher OS and RFS observed in SHCC patients treated by surgical interventions. However, the rates of complications such as bleeding, infection, and liver failure in patients subjected to surgical therapy are high. This finding is consistent with results from previous meta-analyses [92–94]. SR was associated with low recurrence rates and high survival rates, compared to RFA. However, SR was associated with a higher predisposition to severe complications, relative to RFA, although differences were not significant. Currently, there is no unified definition of surgical indications. Previous meta-analyses published in 2010 reported different results [95, 96]. The differences can be attributed to the overall low level of clinical evidence, as most of the studies included in the current study were completed before 2010. Due to advances in interventional radiology in the last decade, loco-regional treatment has become an important alternative therapy for early HCC [17]. The European Association for the Study of the Liver (EASL) recommends RFA and MWA as standard treatment approaches for patients who are not eligible for surgery [6]. MWA uses electromagnetic waves from electrodes, whereas the effects of RFA are achieved by targeting current to the tumor. MWA and RFA are associated with several advantages, such as high tolerance, good repeatability, low complication rates and low initial costs [97–99]. Radiofrequency ablation is associated with some limitations in treatment of SHCC, including the diffusion-thermo effect. Diffusion-thermo effect is attributed to minimum blood vessel flow of 1 ml/min [100], and can lead to incomplete ablation [101]. It has been reported that MWA can alternate conventional radiofrequency ablation, and is highly effective in tumor treatment. A previous meta-analysis indicated that MWA has a lower LTP in larger nodules, compared to RFA [102]. In addition, studies explored complete ablation (CA), local recurrence (LR), PFS, and OS and reported that the efficacies of percutaneous RFA are comparable to those of percutaneous MWA [103]. This study included a high number of samples, performed direct and indirect comparisons, and showed similar outcomes with previous findings [103]. This implies that findings from this study are credible and accurate. In addition, we found that the efficacy and safety of MWA and RFA were comparable, even in < 2 cm or < 3 cm tumor subgroups. It has been documented that CRA has several advantages, including pain relief, immune effects enhancement, and good visualization of ablation areas. Moreover, a larger ablation area can be obtained by simultaneously placing multiple probes. However, due to various safety concerns, this method is not widely used [104]. Due to advances in ablation techniques, the applications of MWA and CRA for SHCC treatment are increasing. Our findings show that CRA exerted comparable efficacies and safety to those of MWA and RFA. Therefore, improvement of the techniques and understanding of their mechanisms can improve the therapeutic effects of existing treatment methods. Notably, the efficacies of PEI and RFA were not significantly different. However, previous studies reported that RFA has a higher effect, compared to PEI [92]. These differences in outcomes could be attributed to a lack of sufficient sample sizes, because only studies published in the past 10 years were included in this meta-analysis. As a result, this meta-analysis only included one study that reported PEI, resulting in a high risk of bias.

A network meta-analysis by Lin et al. [105] analyzed data in 5 RCTs and compared the efficacies of different interventional techniques for treatment of early stage hepatocellular carcinoma, including SR, RFA, MWA, PEI, CRA, laser ablation and external beam radiotherapy. However, in this previous meta-analysis, only RCTs were included, with some high-quality cohort studies being excluded. Furthermore, the study focused on comparing the efficacy of ablation and the studies included in the meta-analysis were published over a large period of time, therefore, technological developments may have resulted in heterogeneity. In addition, the risk ratio (RR) was used as the effect indicator, which may have resulted in errors during survival analysis. A previous NMA compared the efficacies of therapies for SHCC, however, MWA and CRA were not included in the analysis [106]. Moreover, studies published before February 2015 were included in the analysis, with recent studies being excluded. Previous studies recommend the use of SBRT for treatment of HCC that is characterized by relatively large tumors (> 2–3 cm in diameter) as well as for tumors near major vessels or the diaphragm, which is a contraindication for RFA [107–109]. We found that there were no significant differences in efficacies between SBRT and RFA, implying that the effects of confounding factors cannot be completely eliminated. In addition, SBRT is mainly applicable for patients who are not clinically eligible for RFA [109]. These results provide a reference for future research and clinical decision making. However, due to the effects of several confounding factors (such as race, age, facility location, and time of diagnosis), the findings should be treated with caution.

This study has several strengths and a few limitations. i. The strength of this study is that cumulative OS and RFS were compared by calculating HRs (hazard rates), which are the most appropriate parameters for determining time-dependent outcomes [110, 111]. However, HRs were extracted from survival curves, which provided survival information, leading to potential errors. ii. The main limitation for this study is that the included RCTs were few, while most included studies were non-RCTs, resulting in potential unpredictable confounding factors. Notably, the best evidence in oncology is not always based on randomized trials, and reliable data are reported in retrospective studies. In addition, a previous meta-analysis [112] and findings from the study indicated that observational studies mainly produce estimates of effects that are not significantly different from RCTs. Moreover, an important strength of this study is in the overall high methodological quality of the included trials. iii. The current study comprised a large total sample size, however, sample sizes for some treatments were small, implying that some of the findings may not be representative of other populations. Therefore, they should be interpreted with caution. Furthermore, absolute differences among different treatments may be trivial, whereas one treatment may be rated as the best. iv. Further, the study included eight major treatments for SHCC in the analysis. Notably, comparisons of various interventions in SHCC patients may not indicate the benefits of patients from multiple interventions, including combinations of surgical approaches and systemic treatment methods. v. Advances in technology will lead to improved therapeutic effects. Notably, a technology that has been used for a short time may have a disadvantage over a fully developed technology. The current study excluded articles published before 2010, which reduces publication bias to some extent. vi. Data estimated by propensity score matching, and adjusting for potential differences in baseline characteristics of patients was performed to create a highly comparable control group in the meta-analysis. However, most factors were not related to tumor control. Management decisions for SHCC mainly rely on informed preferences of patients and levels of expertise of different medical facilities. vii. Furthermore, we conducted subgroup analysis based on study types, tumor sizes and outcomes. However, due to insufficient data, subgroup analyses or regression analyses were not conducted on some of the factors that may have affected patient outcomes.

## Conclusions

The findings of this network meta-analysis indicated that MIS and SR exhibit high clinical efficacies, however, these two approaches are correlated with a high number of complications. Ablation is highly effective for small tumors, whereas SBRT is more effective when compared to other ablation treatments in some cases. This indicates that SBRT is a relatively promising treatment for HCC. Subgroup analysis indicated that further studies should explore indications for different treatments. Moreover, well-designed, large-scale randomized controlled trials should be conducted to validate the findings of this study.

## Supplementary Information

**Additional file 1**: Figure S1: Quality assessment of included RCTs using Cochrane risk of bias assessment tool. RCTs: randomized controlled trials.

**Additional file 2**:** Figure S2**. Funnel plot showing standard error by RR for major complication rates. RR: risk ratio.

**Additional file 3**:** Figure S3**. Results on convergence of Gelman Rubin diagnostics. A: Results on OS; B: Results on RFS* (RFS, PFS, DFS and TFS were combined and redefined as RFS*); C: Results on major complications rate. 1: RFA, 2: MWA, 3: SR, 4: TACE, 5: PEI, 6: MIS, 7: SBRT, 8: CRA. The level of adequacy of convergence of Gelman Rubin diagnostics approached 1 for all the three outcome parameters, indicating good convergence. OS: Overall Survival, RFS: recurrence-free survival, PFS: progression-free survival, DFS: disease free survival, TFS, tumor-free survival, RFA: radiofrequency ablation, MWA: microwave ablation, SR: surgical resection, TACE: transarterial chemoembolization, PEI: percutaneous ethanol injection, MIS: Minimally invasive liver surgery, SBRT: stereotactic body radiotherapy, CRA: cryotherapy ablation.

**Additional file 4**:** Figure S4**. Forest plots showing relationships between different interventional methods and OS for subgroup analyses (HCCs tumor size ≤ 5 cm), compared to RFA and MWA. HR values and 95% CI were used. 1: RFA, 2: MWA, 3: SR, 6: MIS, 7: SBRT, 8: CRA. OS: Overall Survival, HCC: Hepatocellular Carcinoma, RFA: radiofrequency ablation, MWA: microwave ablation, HR: Hazard ratio, SR: surgical resection, MIS: Minimally invasive liver surgery, SBRT: stereotactic body radiotherapy, CRA: cryotherapy ablation.

**Additional file 5**:** Figure S5**. Forest plots showing the relationship between different interventional methods and OS for subgroup analyses (HCCs tumor size ≤ 3 cm) compared to RFA. HR values and 95% CI were used for comparisons. 1: RFA, 3: SR, 4: TACE, 5: PEI, 6: MIS, 7: SBRT. OS: Overall Survival, HCC: Hepatocellular carcinoma, RFA: radiofrequency ablation, HR: Hazard ratio, SR: surgical resection, TACE: transarterial chemoembolization, PEI: percutaneous ethanol injection, MIS: Minimally invasive liver surgery, SBRT: stereotactic body radiotherapy.

**Additional file 6**:** Figure S6**. Forest plots showing the relationship between different interventional methods and OS for subgroup analyses (RCTs) compared to RFA. HR values and 95% CI were used for comparisons. 1: RFA, 2: MWA, 3: SR, 5: PEI, 8: CRA. OS: Overall Survival, RCTs: randomized controlled trials, RFA: radiofrequency ablation, HR: Hazard ratio, MWA: microwave ablation, SR: surgical resection, PEI: percutaneous ethanol injection, CRA: cryotherapy ablation.

**Additional file 7**:** Figure S7**. Forest plots showing the relationship between different interventional approaches and OS for subgroup analyses (non-RCTs), compared to RFA and MWA. HR values and 95% CI were used for comparisons. 1: RFA, 2: MWA, 3: SR, 4: TACE, 6: MIS, 7: SBRT, 8: CRA. OS: Overall Survival, non-RCTs: non-randomized controlled trials, RFA: radiofrequency ablation, MWA: microwave ablation, HR: Hazard ratio, SR: surgical resection, TACE: transarterial chemoembolization, MIS: Minimally invasive liver surgery, SBRT: stereotactic body radiotherapy, CRA: cryotherapy ablation.

**Additional file 8**:** Figure S8**. Forest plots showing the relationship between different interventional methods and DFS, compared to RFA and MWA. HR values and 95% CI were used for comparisons. 1: RFA, 2: MWA, 3: SR, 6: MIS, 7: SBRT. DFS: disease free survival, RFA: radiofrequency ablation, MWA: microwave ablation, HR: Hazard ratio, CI: confidence interval, SR: surgical resection, MIS: Minimally invasive liver surgery, SBRT: stereotactic body radiotherapy.

**Additional file 9**:** Figure S9**. Forest plots showing the relationship between different interventional methods and RFS, compared to RFA, MWA and CRA. HR values and 95% CI were used for comparisons. 1: RFA, 2: MWA, 3: SR, 4: TACE, 6: MIS, 7: SBRT, 8: CRA. RFS: recurrence-free survival, RFA: radiofrequency ablation, MWA: microwave ablation, HR: Hazard ratio, CI: confidence interval, SR: surgical resection, TACE: transarterial chemoembolization, MIS: Minimally invasive liver surgery, SBRT: stereotactic body radiotherapy.

**Additional file 10**:** Figure S10**. Forest plots showing the association between different interventional methods and RFS* (RFS, PFS, DFS and TFS were combined and redefined as RFS*) for subgroup analyses (RCTs), compared to RFA. HR values and 95% CI were used for comparisons. 1: RFA, 2: MWA, 3: SR, 8: CRA. RFS: recurrence-free survival, PFS: progression-free survival, DFS: disease free survival, TFS, tumor-free survival, RCTs: randomized controlled trials, RFA: radiofrequency ablation, HR: Hazard ratio, CI: confidence interval, MWA: microwave ablation, SR: surgical resection, CRA: cryotherapy ablation.

**Additional file 11**:** Figure S11**. Forest plots showing the association between different interventional methods and RFS* (RFS, PFS, DFS and TFS were combined and redefined as RFS*) for subgroup analyses (non-RCTs), compared to RFA and MWA. HR values and 95% CI were used for comparisons. 1: RFA, 2: MWA, 3: SR, 4: TACE, 6: MIS, 7: SBRT, 8: CRA. RFS: recurrence-free survival, PFS: progression-free survival, DFS: disease free survival, TFS, tumor-free survival, non-RCTs: non-randomized controlled trials, RFA: radiofrequency ablation, MWA: microwave ablation, HR: Hazard ratio, CI: confidence interval, SR: surgical resection, TACE: transarterial chemoembolization, MIS: Minimally invasive liver surgery, SBRT: stereotactic body radiotherapy, CRA: cryotherapy ablation.

**Additional file 12**:** Figure S12**. Forest plots showing the association between different interventional arms and RFS* (RFS, PFS, DFS and TFS were combined and redefined as RFS*) for subgroup analyses (HCCs tumor size ≤ 5 cm), compared to RFA and MWA. HR values and 95% CI were used for comparisons. 1: RFA, 2: MWA, 3: SR, 6: MIS, 7: SBRT, 8: CRA. RFS: recurrence-free survival, PFS: progression-free survival, DFS: disease free survival, TFS, tumor-free survival, HCC: Hepatocellular Carcinoma, RFA: radiofrequency ablation, MWA: microwave ablation, HR: Hazard ratio, CI: confidence interval, SR: surgical resection, MIS: Minimally invasive liver surgery, SBRT: stereotactic body radiotherapy, CRA: cryotherapy ablation.

**Additional file 13**:** Figure S13**. Forest plots showing the association between different interventional approaches and RFS* (RFS, PFS, DFS and TFS were combined and redefined as RFS*) for subgroup analyses (HCCs tumor size ≤ 3 cm), compared to TACE. HR values and 95% CI were used for comparisons. 1: RFA, 3: SR, 4: TACE, 6: MIS. RFS: recurrence-free survival, PFS: progression-free survival, DFS: disease free survival, TFS, tumor-free survival, HCC: Hepatocellular Carcinoma, TACE: transarterial chemoembolization, HR: Hazard ratio, CI: confidence interval, RFA: radiofrequency ablation, SR: surgical resection, MIS: Minimally invasive liver surgery.

**Additional file 14**:** Figure S14**. Forest plots showing the association between different interventional methods and major complication rates in subgroup analyses (RCTs), compared to RFA. RR values and 95% CI were used for comparisons. 1: RFA, 2: MWA, 3: SR, 5: PEI, 8: CRA. RCTs: randomized controlled trials, RFA: radiofrequency ablation, RR: risk ratio, MWA: microwave ablation, SR: surgical resection, PEI: percutaneous ethanol injection, CRA: cryotherapy ablation.

**Additional file 15**:** Figure S15**. Forest plots showing the association between different interventional approaches and major complications rate for subgroup analyses (non-RCTs), compared to RFA. RR values and 95% CI were used for comparisons. 1: RFA, 2: MWA, 3: SR, 4: TACE, 6: MIS, 7: SBRT, 8: CRA. non-RCTs: non-randomized controlled trials, RFA: radiofrequency ablation, RR: risk ratio, MWA: microwave ablation, SR: surgical resection, TACE: transarterial chemoembolization, MIS: Minimally invasive liver surgery, SBRT: stereotactic body radiotherapy, CRA: cryotherapy ablation.

**Additional file 15**: **Table S1**. The inconsistent loops P-value for different comparisons.

## Data Availability

Not applicable.
