# Supplementary figures and images for: Efficacy and safety of various primary treatment strategies for very early and early hepatocellular carcinoma: a network meta-analysis
Source: Cancer Cell Int. 2021 Dec 19;21:681. doi: 10.1186/s12935-021-02365-1 (PMC8684647; doi:10.1186/s12935-021-02365-1)

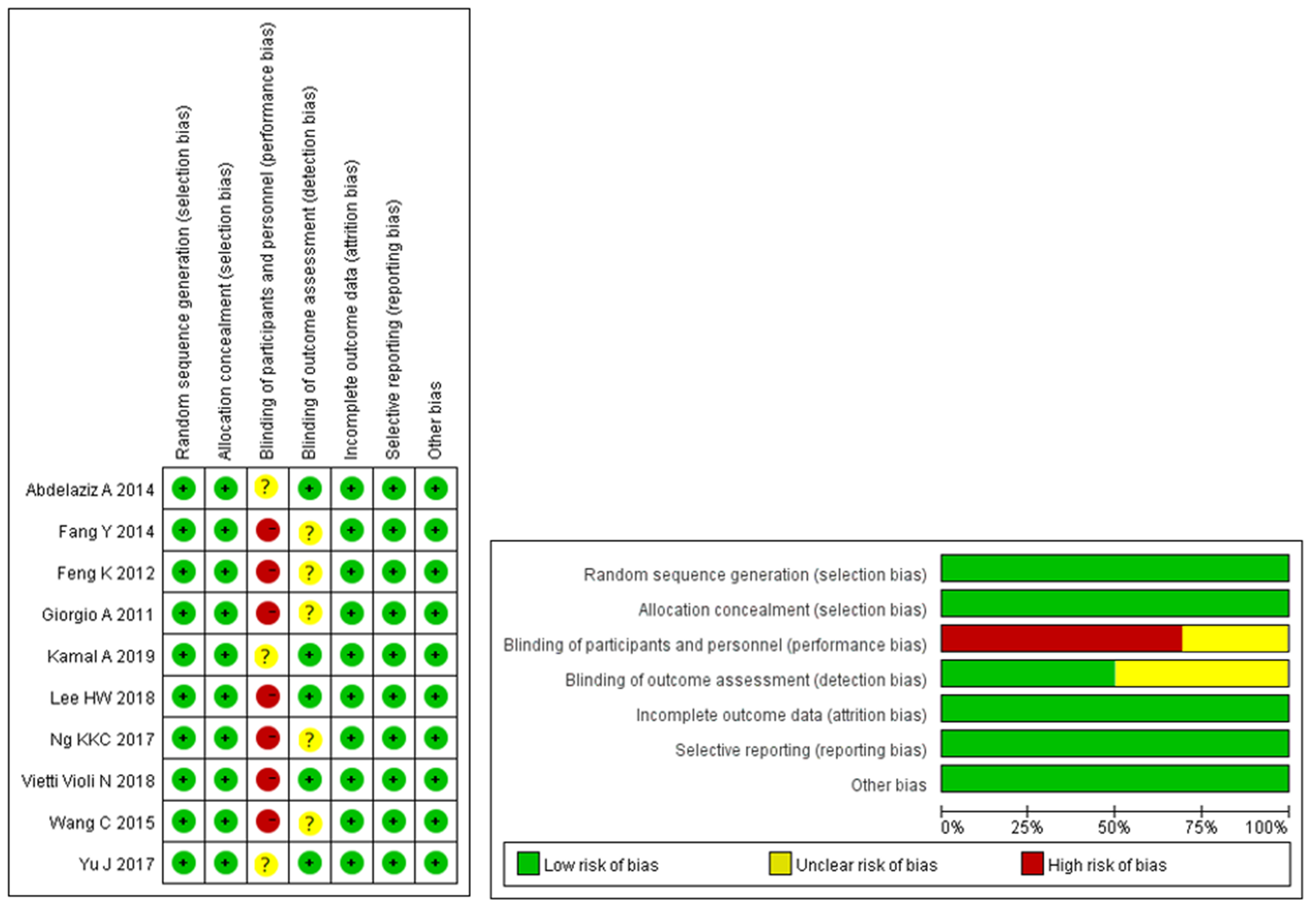

Supplement: Supplementary file 1 — Additional file 1: Figure S1: Quality assessment of included RCTs using Cochrane risk of bias assessment tool. RCTs: randomized controlled trials. [file 12935_2021_2365_MOESM1_ESM.tif]

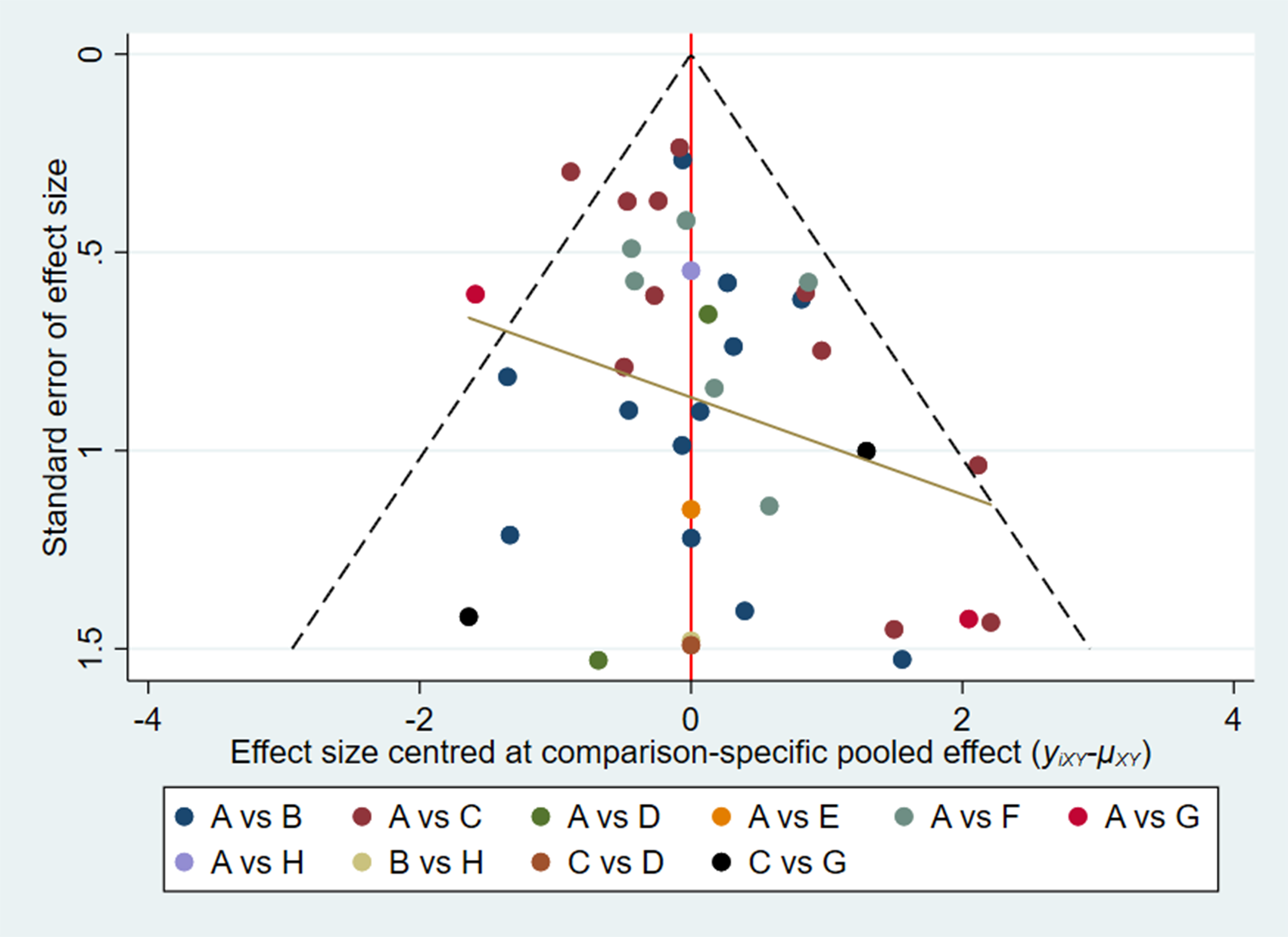

Supplement: Supplementary file 2 — Additional file 2: Figure S2. Funnel plot showing standard error by RR for major complication rates. RR: risk ratio. [file 12935_2021_2365_MOESM2_ESM.tif]

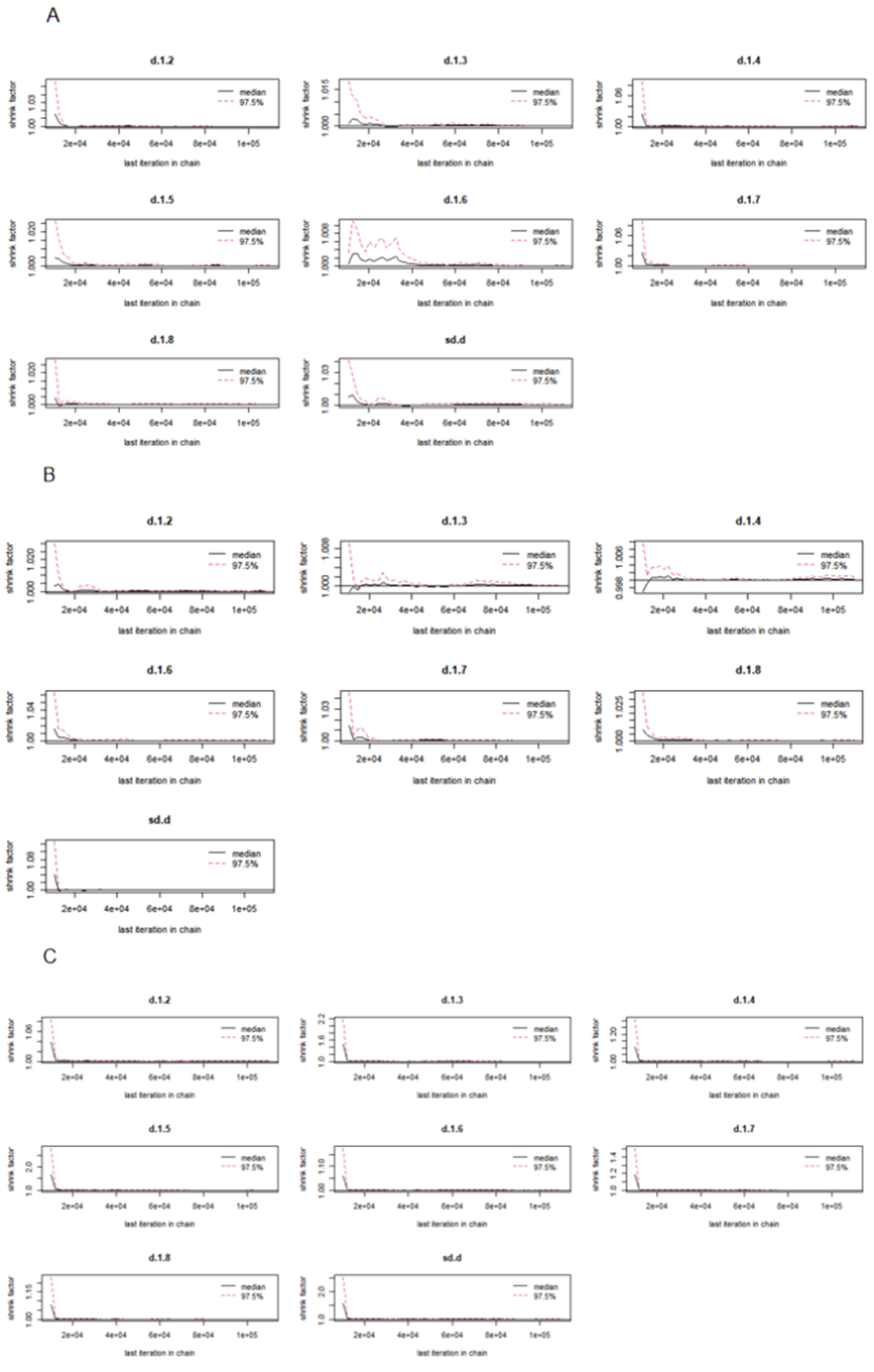

Supplement: Supplementary file 3 — Additional file 3: Figure S3. Results on convergence of Gelman Rubin diagnostics. A: Results on OS; B: Results on RFS* (RFS, PFS, DFS and TFS were combined and redefined as RFS*); C: Results on major complications rate. 1: RFA, 2: MWA, 3: SR, 4: TACE, 5: PEI, 6: MIS, 7: SBRT, 8: CRA. The level of adequacy of convergence of Gelman Rubin diagnostics approached 1 for all the three outcome parameters, indicating good convergence. OS: Overall Survival, RFS: recurrence-free survival, PFS: progression-free survival, DFS: disease free survival, TFS, tumor-free survival, RFA: radiofrequency ablation, MWA: microwave ablation, SR: surgical resection, TACE: transarterial chemoembolization, PEI: percutaneous ethanol injection, MIS: Minimally invasive liver surgery, SBRT: stereotactic body radiotherapy, CRA: cryotherapy ablation. [file 12935_2021_2365_MOESM3_ESM.tif]

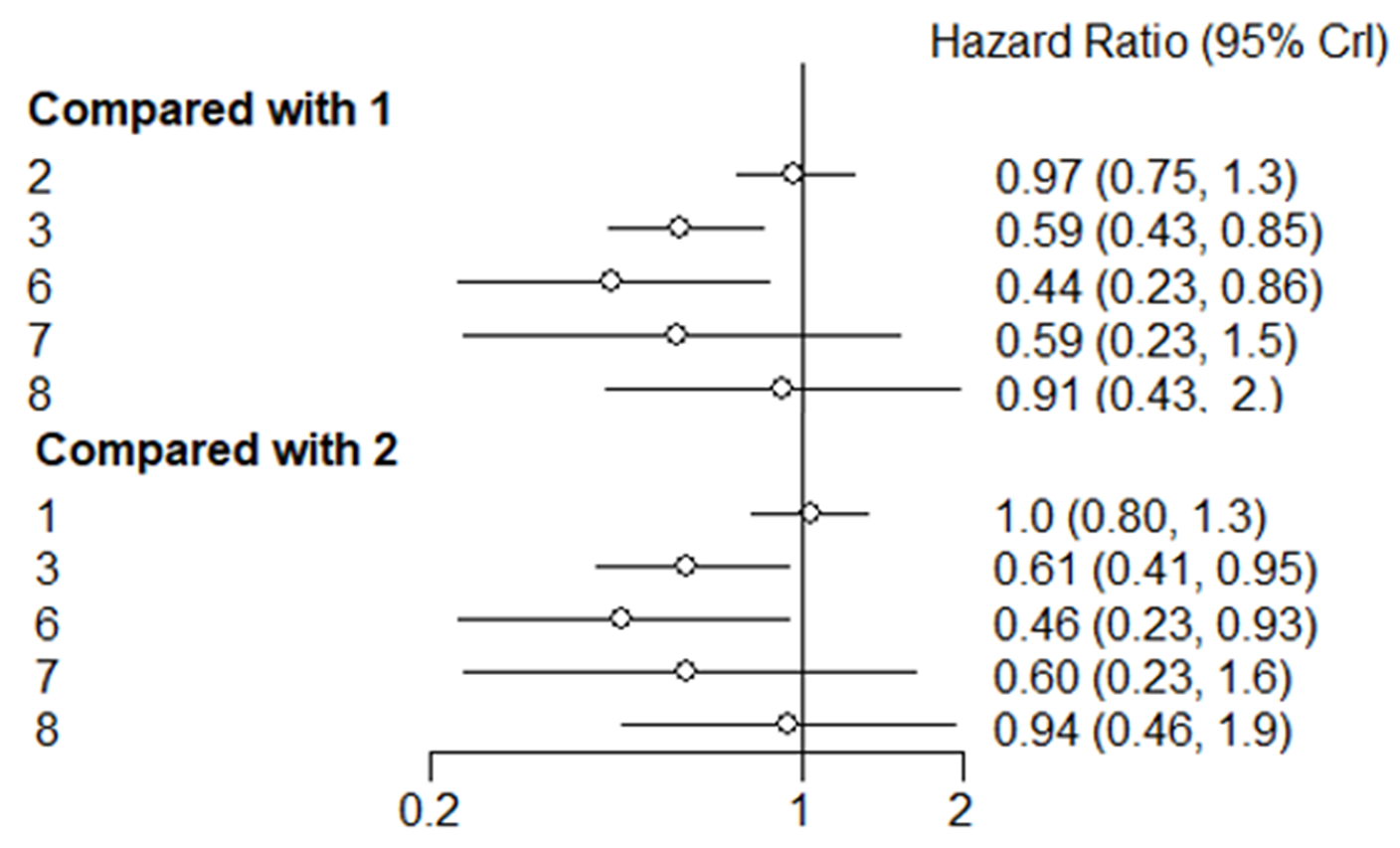

Supplement: Supplementary file 4 — Additional file 4: Figure S4. Forest plots showing relationships between different interventional methods and OS for subgroup analyses (HCCs tumor size ≤ 5 cm), compared to RFA and MWA. HR values and 95% CI were used. 1: RFA, 2: MWA, 3: SR, 6: MIS, 7: SBRT, 8: CRA. OS: Overall Survival, HCC: Hepatocellular Carcinoma, RFA: radiofrequency ablation, MWA: microwave ablation, HR: Hazard ratio, SR: surgical resection, MIS: Minimally invasive liver surgery, SBRT: stereotactic body radiotherapy, CRA: cryotherapy ablation. [file 12935_2021_2365_MOESM4_ESM.tif]

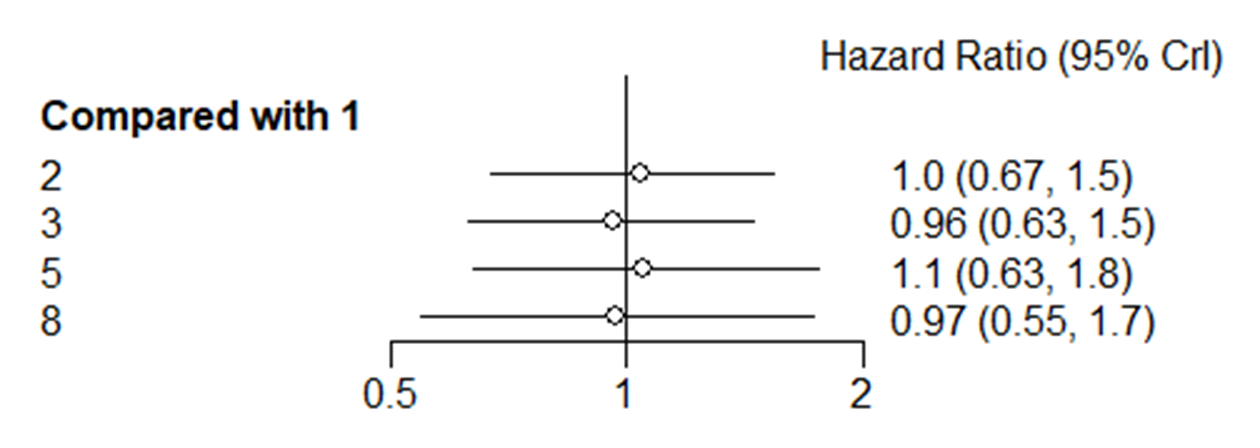

Supplement: Supplementary file 6 — Additional file 6: Figure S6. Forest plots showing the relationship between different interventional methods and OS for subgroup analyses (RCTs) compared to RFA. HR values and 95% CI were used for comparisons. 1: RFA, 2: MWA, 3: SR, 5: PEI, 8: CRA. OS: Overall Survival, RCTs: randomized controlled trials, RFA: radiofrequency ablation, HR: Hazard ratio, MWA: microwave ablation, SR: surgical resection, PEI: percutaneous ethanol injection, CRA: cryotherapy ablation. [file 12935_2021_2365_MOESM6_ESM.tif]

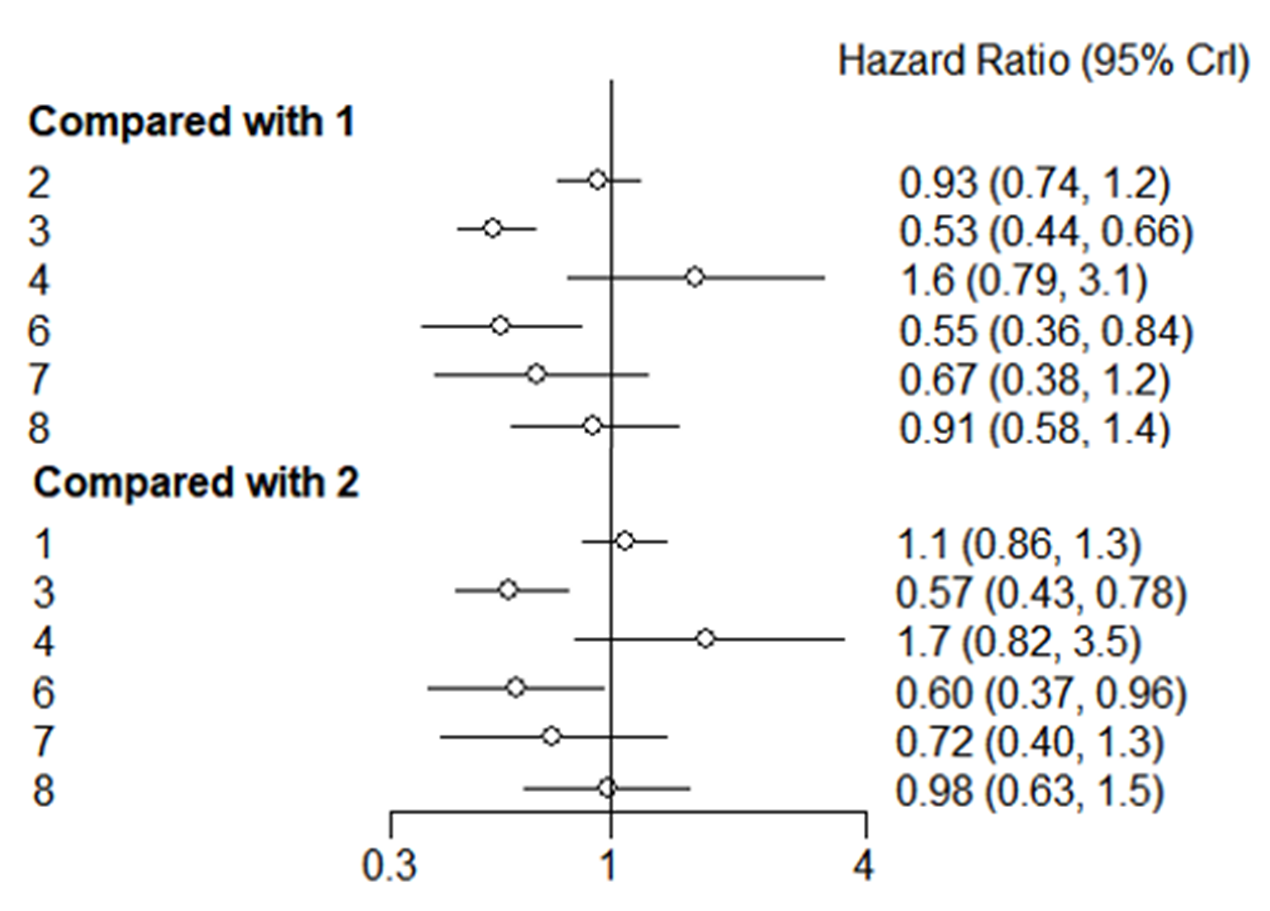

Supplement: Supplementary file 7 — Additional file 7: Figure S7. Forest plots showing the relationship between different interventional approaches and OS for subgroup analyses (non-RCTs), compared to RFA and MWA. HR values and 95% CI were used for comparisons. 1: RFA, 2: MWA, 3: SR, 4: TACE, 6: MIS, 7: SBRT, 8: CRA. OS: Overall Survival, non-RCTs: non-randomized controlled trials, RFA: radiofrequency ablation, MWA: microwave ablation, HR: Hazard ratio, SR: surgical resection, TACE: transarterial chemoembolization, MIS: Minimally invasive liver surgery, SBRT: stereotactic body radiotherapy, CRA: cryotherapy ablation. [file 12935_2021_2365_MOESM7_ESM.tif]

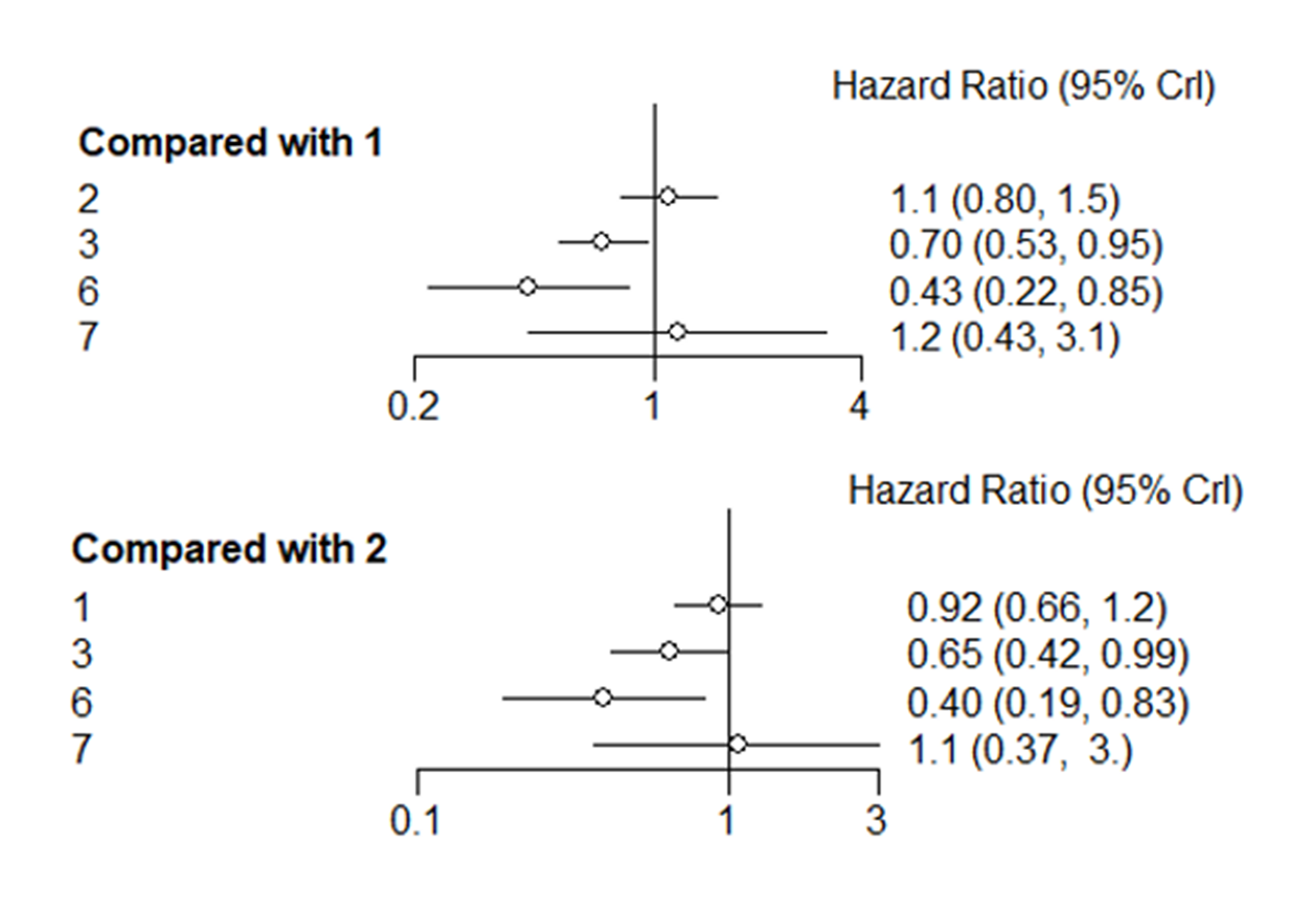

Supplement: Supplementary file 8 — Additional file 8: Figure S8. Forest plots showing the relationship between different interventional methods and DFS, compared to RFA and MWA. HR values and 95% CI were used for comparisons. 1: RFA, 2: MWA, 3: SR, 6: MIS, 7: SBRT. DFS: disease free survival, RFA: radiofrequency ablation, MWA: microwave ablation, HR: Hazard ratio, CI: confidence interval, SR: surgical resection, MIS: Minimally invasive liver surgery, SBRT: stereotactic body radiotherapy. [file 12935_2021_2365_MOESM8_ESM.tif]

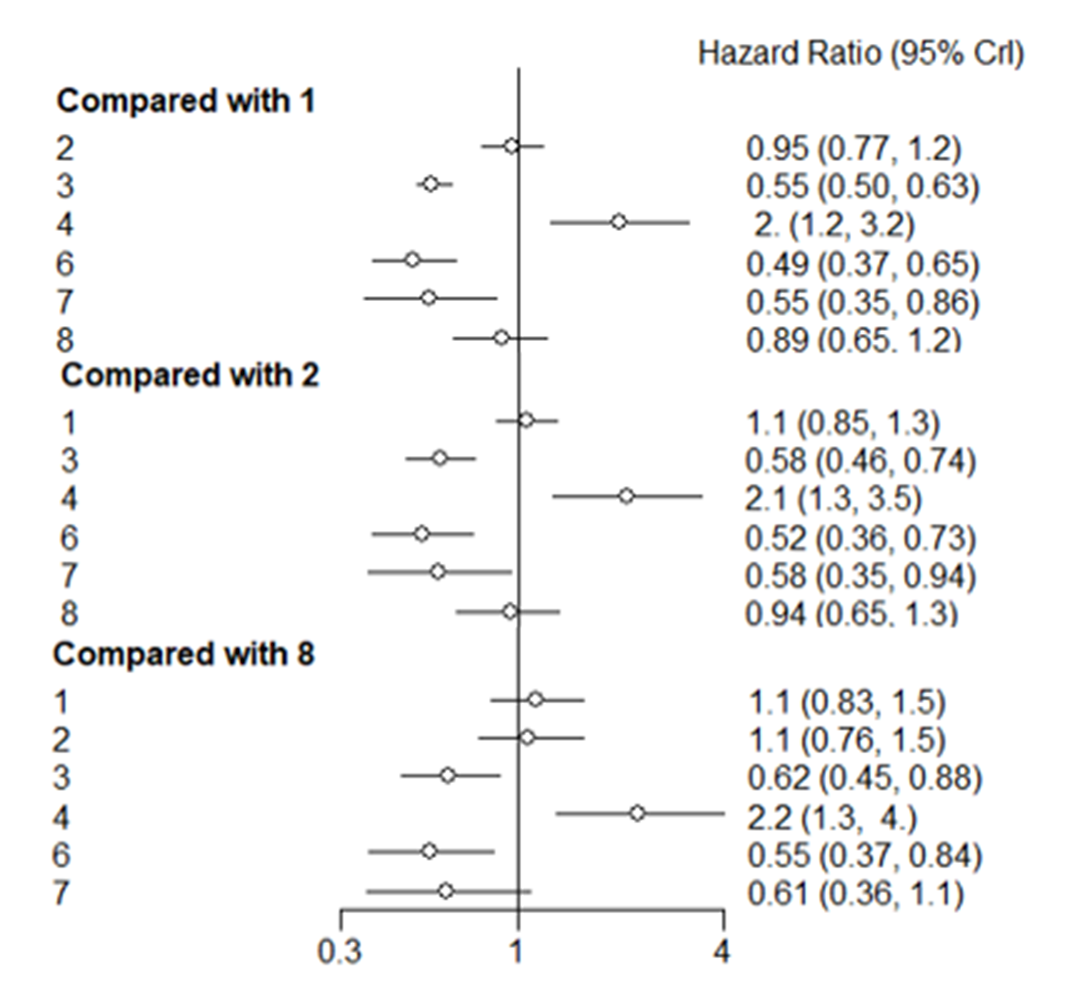

Supplement: Supplementary file 9 — Additional file 9: Figure S9. Forest plots showing the relationship between different interventional methods and RFS, compared to RFA, MWA and CRA. HR values and 95% CI were used for comparisons. 1: RFA, 2: MWA, 3: SR, 4: TACE, 6: MIS, 7: SBRT, 8: CRA. RFS: recurrence-free survival, RFA: radiofrequency ablation, MWA: microwave ablation, HR: Hazard ratio, CI: confidence interval, SR: surgical resection, TACE: transarterial chemoembolization, MIS: Minimally invasive liver surgery, SBRT: stereotactic body radiotherapy. [file 12935_2021_2365_MOESM9_ESM.tif]

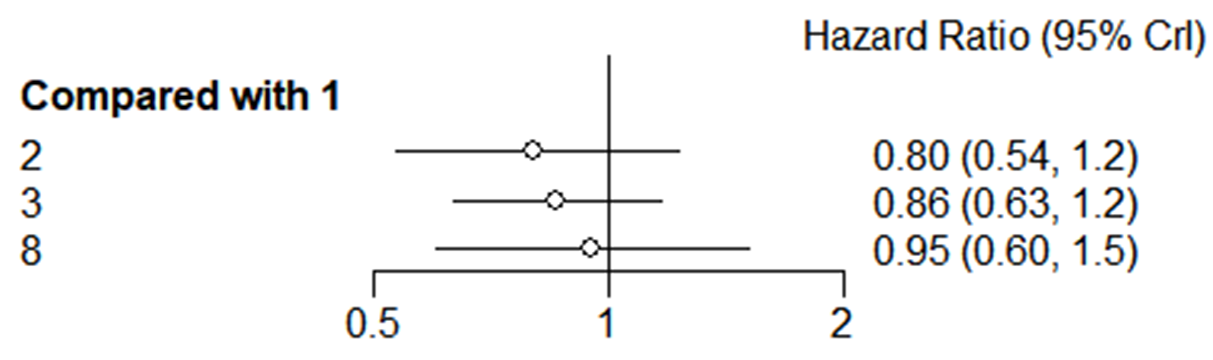

Supplement: Supplementary file 10 — Additional file 10: Figure S10. Forest plots showing the association between different interventional methods and RFS* (RFS, PFS, DFS and TFS were combined and redefined as RFS*) for subgroup analyses (RCTs), compared to RFA. HR values and 95% CI were used for comparisons. 1: RFA, 2: MWA, 3: SR, 8: CRA. RFS: recurrence-free survival, PFS: progression-free survival, DFS: disease free survival, TFS, tumor-free survival, RCTs: randomized controlled trials, RFA: radiofrequency ablation, HR: Hazard ratio, CI: confidence interval, MWA: microwave ablation, SR: surgical resection, CRA: cryotherapy ablation. [file 12935_2021_2365_MOESM10_ESM.tif]

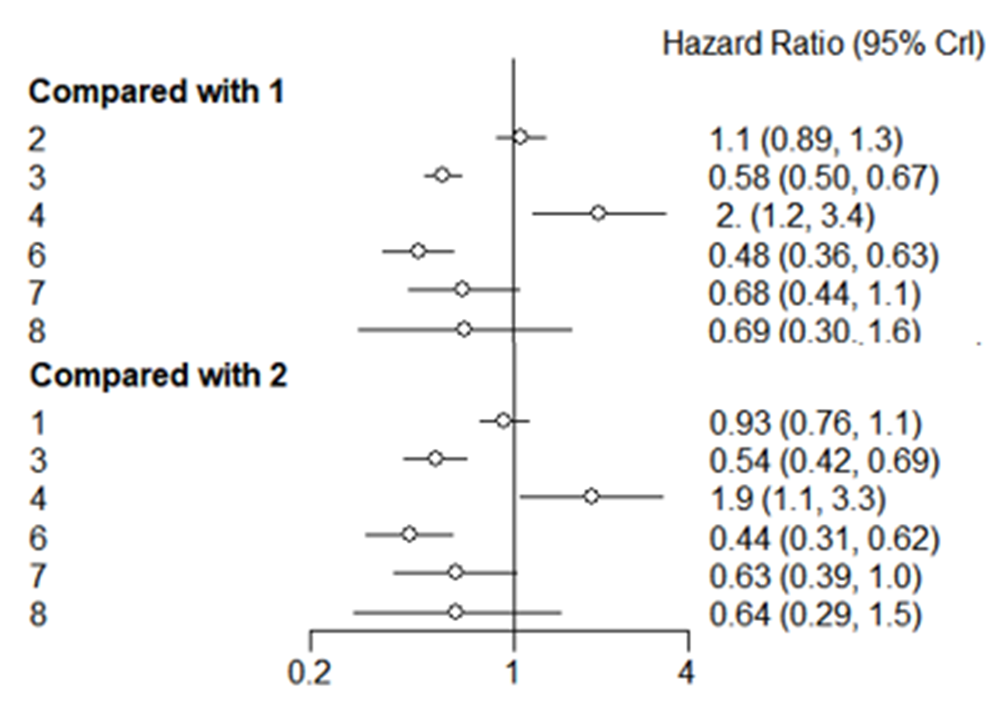

Supplement: Supplementary file 11 — Additional file 11: Figure S11. Forest plots showing the association between different interventional methods and RFS* (RFS, PFS, DFS and TFS were combined and redefined as RFS*) for subgroup analyses (non-RCTs), compared to RFA and MWA. HR values and 95% CI were used for comparisons. 1: RFA, 2: MWA, 3: SR, 4: TACE, 6: MIS, 7: SBRT, 8: CRA. RFS: recurrence-free survival, PFS: progression-free survival, DFS: disease free survival, TFS, tumor-free survival, non-RCTs: non-randomized controlled trials, RFA: radiofrequency ablation, MWA: microwave ablation, HR: Hazard ratio, CI: confidence interval, SR: surgical resection, TACE: transarterial chemoembolization, MIS: Minimally invasive liver surgery, SBRT: stereotactic body radiotherapy, CRA: cryotherapy ablation. [file 12935_2021_2365_MOESM11_ESM.tif]

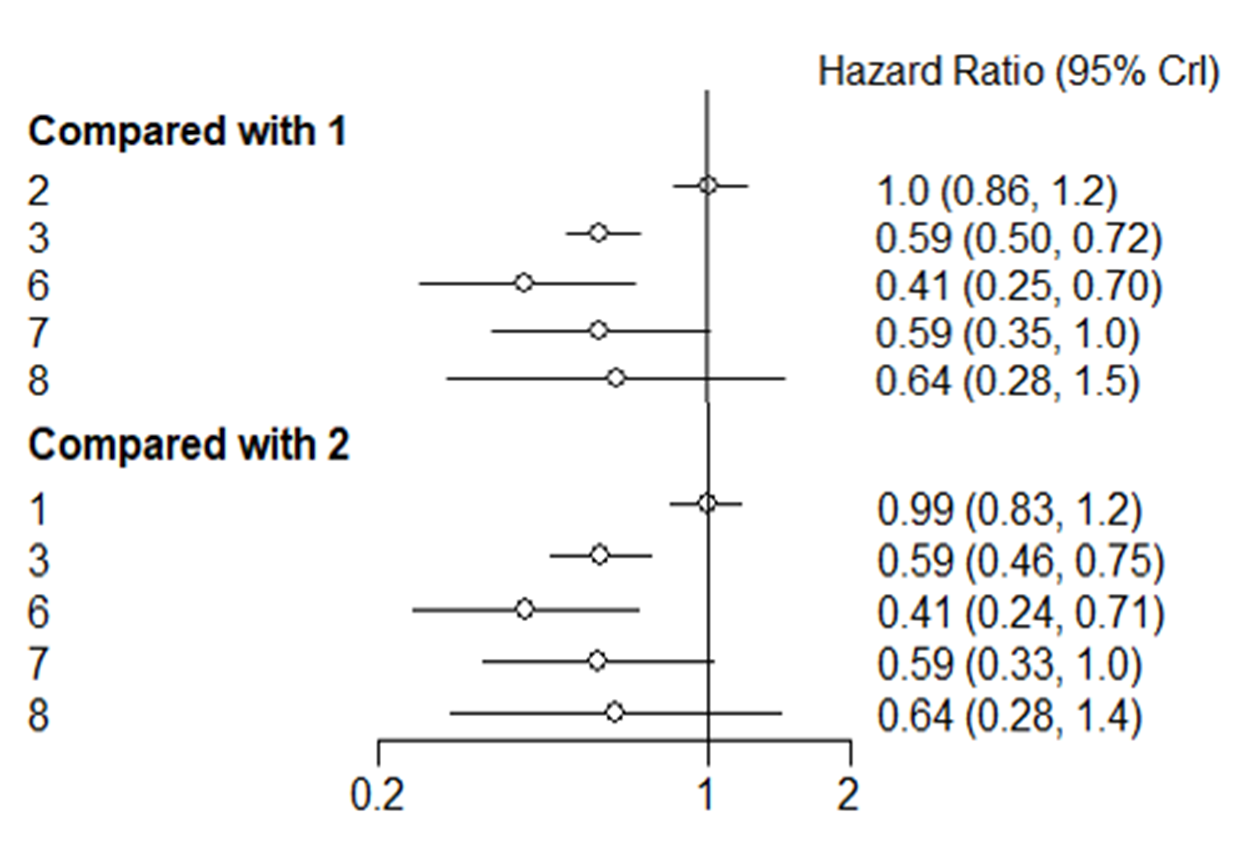

Supplement: Supplementary file 12 — Additional file 12: Figure S12. Forest plots showing the association between different interventional arms and RFS* (RFS, PFS, DFS and TFS were combined and redefined as RFS*) for subgroup analyses (HCCs tumor size ≤ 5 cm), compared to RFA and MWA. HR values and 95% CI were used for comparisons. 1: RFA, 2: MWA, 3: SR, 6: MIS, 7: SBRT, 8: CRA. RFS: recurrence-free survival, PFS: progression-free survival, DFS: disease free survival, TFS, tumor-free survival, HCC: Hepatocellular Carcinoma, RFA: radiofrequency ablation, MWA: microwave ablation, HR: Hazard ratio, CI: confidence interval, SR: surgical resection, MIS: Minimally invasive liver surgery, SBRT: stereotactic body radiotherapy, CRA: cryotherapy ablation. [file 12935_2021_2365_MOESM12_ESM.tif]

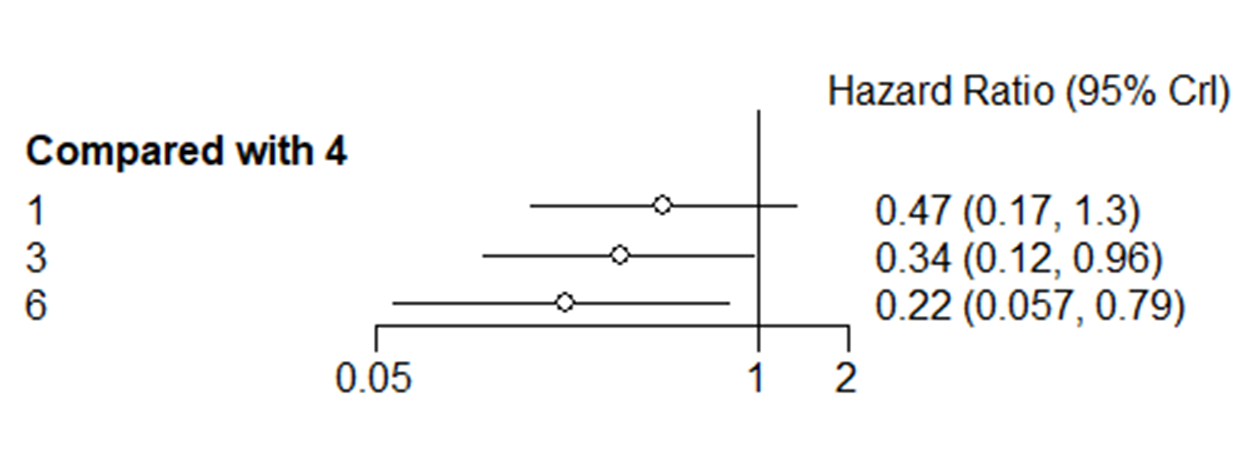

Supplement: Supplementary file 13 — Additional file 13: Figure S13. Forest plots showing the association between different interventional approaches and RFS* (RFS, PFS, DFS and TFS were combined and redefined as RFS*) for subgroup analyses (HCCs tumor size ≤ 3 cm), compared to TACE. HR values and 95% CI were used for comparisons. 1: RFA, 3: SR, 4: TACE, 6: MIS. RFS: recurrence-free survival, PFS: progression-free survival, DFS: disease free survival, TFS, tumor-free survival, HCC: Hepatocellular Carcinoma, TACE: transarterial chemoembolization, HR: Hazard ratio, CI: confidence interval, RFA: radiofrequency ablation, SR: surgical resection, MIS: Minimally invasive liver surgery. [file 12935_2021_2365_MOESM13_ESM.tif]

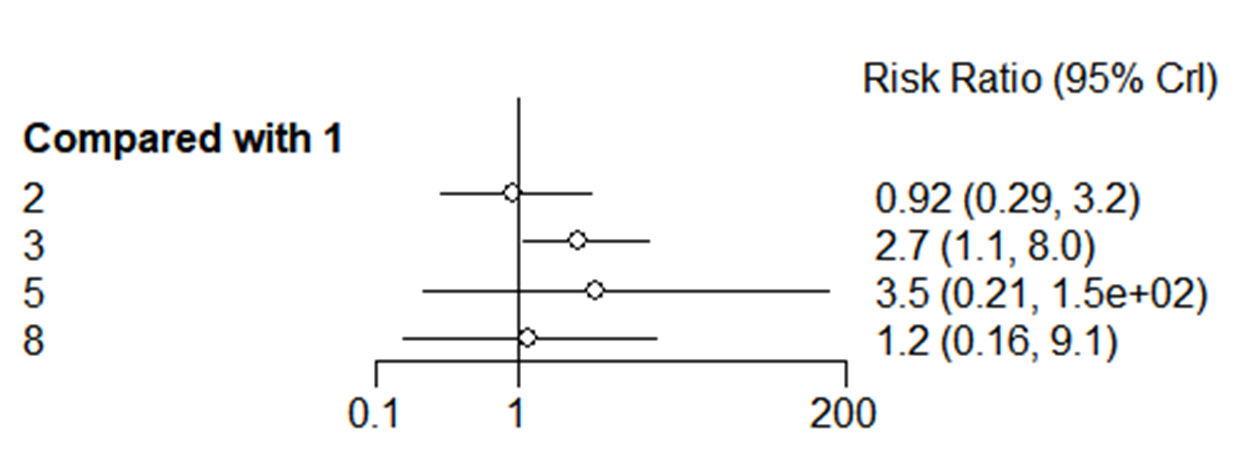

Supplement: Supplementary file 14 — Additional file 14: Figure S14. Forest plots showing the association between different interventional methods and major complication rates in subgroup analyses (RCTs), compared to RFA. RR values and 95% CI were used for comparisons. 1: RFA, 2: MWA, 3: SR, 5: PEI, 8: CRA. RCTs: randomized controlled trials, RFA: radiofrequency ablation, RR: risk ratio, MWA: microwave ablation, SR: surgical resection, PEI: percutaneous ethanol injection, CRA: cryotherapy ablation. [file 12935_2021_2365_MOESM14_ESM.tif]

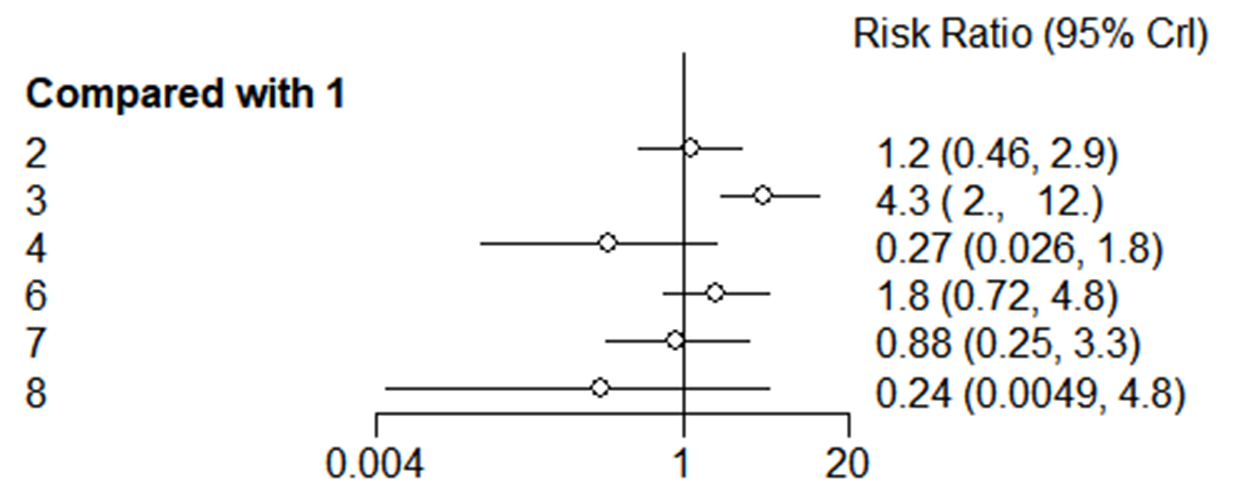

Supplement: Supplementary file 15 — Additional file 15: Figure S15. Forest plots showing the association between different interventional approaches and major complications rate for subgroup analyses (non-RCTs), compared to RFA. RR values and 95% CI were used for comparisons. 1: RFA, 2: MWA, 3: SR, 4: TACE, 6: MIS, 7: SBRT, 8: CRA. non-RCTs: non-randomized controlled trials, RFA: radiofrequency ablation, RR: risk ratio, MWA: microwave ablation, SR: surgical resection, TACE: transarterial chemoembolization, MIS: Minimally invasive liver surgery, SBRT: stereotactic body radiotherapy, CRA: cryotherapy ablation. [file 12935_2021_2365_MOESM15_ESM.tif]
