# Supplementary material for: Efficacy and safety of various primary treatment strategies for very early and early hepatocellular carcinoma: a network meta-analysis
Source: Cancer Cell Int. 2021 Dec 19;21:681. doi: 10.1186/s12935-021-02365-1 (PMC8684647; doi:10.1186/s12935-021-02365-1)
Supplement: Supplementary file 16 — Additional file 15: Table S1. The inconsistent loops P-value for different comparisons. [file 12935_2021_2365_MOESM16_ESM.doc]

Supplementary table 1. The inconsistent loops P-value for different comparisons.

|  | 2 vs 1 | 3 vs 1 | 4 vs 1 | 7 vs 1 | 8 vs 1 | 8 vs 2 | 4 vs 3 | 6 vs 3 | 7 vs 3 |
| --- | --- | --- | --- | --- | --- | --- | --- | --- | --- |
| OS | 0.96 | 0.88 |  | 0.89 | 0.96 | 0.96 | 0.46 |  | 0.89 |
| RFS* | 0.43 | 0.83 |  | 0.84 | 0.44 | 0.44 |  | 0.53 | 0.84 |
| C | 0.32 | 0.33 | 0.35 | 0.33 | 0.32 | 0.32 | 0.34 |  | 0.33 |

1: RFA, 2: MWA, 3:SR, 4:TACE, 5:PEI, 6:MIS, 7:SBRT, 8:CRA, C: major complications rate. P＞ 0.05 indicates no loop inconsistencie.
